# High molecular weight insoluble parkin in the substantia nigra of patients with idiopathic Parkinson’s disease

**DOI:** 10.1038/s41531-026-01371-2

**Published:** 2026-05-07

**Authors:** Cyntia Tremblay, Laura Pshevorskiy, Rosalie J. Cottez, Hélèna L. Denis, Vincent Emond, Marc Morissette, Ali H. Rajput, Thérèse Di Paolo, Alex Rajput, Frédéric Calon

**Affiliations:** 1 Neuroscience Research program, CHU de Québec-Université Laval Research center, Québec, Canada; 2 Faculté de pharmacie, Université Laval, Québec, Canada; 3 Movement Disorders Program, University of Saskatchewan, Saskatoon, SK Canada; 4 Faculty of Medicine, University of Saskatchewan, Saskatoon, SK Canada

**Keywords:** Diseases, Neurology, Neuroscience

## Abstract

Parkinson’s disease (PD) is characterized by a loss of dopaminergic neurons and accumulation of α-synuclein (α-syn)-containing Lewy bodies in the substantia nigra (SN) pars compacta. Mutations in the gene coding for the protein parkin cause a form of autosomal recessive juvenile parkinsonism, but its role in idiopathic PD is poorly understood. Here, to investigate parkin changes in the SN in PD, we established a clinicopathology research platform comparing PD patients (*n* = 24) with Controls (*n* = 21). We first confirmed the massive loss of dopamine (DA) levels (−96%) in the putamen of PD patients, using HPLC/electrochemistry. Higher levels of phosphorylated α-syn (αsynP129) (23-fold) were observed in the SN of PD patients by Western immunoblotting. In formic acid extracts, an increase in the insoluble oligomeric form of parkin migrating at 260 kDa was observed (+ 49%) in the SN of PD patients, along with lower levels of the 55 kDa monomeric form (−47%). These changes in parkin were specific for the SN, and not observed in the putamen, parietal cortex and cerebellum. High molecular weight parkin correlated with αsynP129 levels and dopamine loss and was more prominently found in PD patients with levodopa-induced dyskinesias. Additional studies in animal models suggest that the aggregation of parkin is not a direct consequence of dopaminergic depletion or αsyn overproduction, but a component of PD cellular pathophysiology. Taken together, the results reported herein show that, beside dopamine loss and increased αsynP129, neurodegeneration in idiopathic PD is associated with a conversion of parkin into an insoluble high molecular weight form in the SN.

## Introduction

Parkinson’s disease (PD) is a progressive neurodegenerative disorder firstly characterized by the loss of dopaminergic neurons in the substantia nigra (SN) pars compacta (SNpc). This results in a massive decrease in the dopamine levels in the presynaptic terminals estimated to be between 60 and 99% in caudate and putamen of patients^1–4^. Restoring dopamine function in the basal ganglia remains the mainstay of clinical therapeutic interventions^5–7^. In the last two decades, the PD research field has shifted its focus away from dopamine towards considering PD as an α-synuclein (αsyn) proteinopathy This view is chiefly based on genetic data and the observed presence of αsyn in Lewy bodies in the SN of PD patients^5,8–10^. The phosphorylation at serine 129 (αsynP129) is closely associated with abnormal αsyn aggregation^11–14^. Duplication and triplication of the wild-type αsyn gene suffice to cause parkinsonism, suggesting that higher concentrations of the αsyn protein may be involved in sporadic PD^15^. From this came the hypothesis that simply reducing αsyn levels could be therapeutic^9,16^, a view that has been recently disputed^17–20^. Unfortunately, it must be recognized that, contrary to hypotheses based on dopamine loss, the focus on αsyn has not been particularly successful in yielding new therapeutic options for PD patients. Therefore, after more than 50 years following the discovery of dopamine loss in PD, the need to decipher new pathognomonic signs of PD within the SN remains dire.

Genetic advances highlight the multifactorial and polygenic nature of PD^21–23^. In particular, the loss of function of the ubiquitin E3 ligase parkin (aka PRKN) has been shown to cause genetic forms of PD^24–26^. PRKN variants are the most common cause of autosomal recessive PD, accounting for > 40% of the familial early-onset cases^24,27,28^. Following activation by PINK1, parkin exerts a control on protein degradation, mitochondrial homeostasis and cellular mitophagy^24,29,30^. Parkin can undergo post-translational modifications (PTMs) such as phosphorylation, ubiquitination, sumoylation and neddylation as well nitrosylation, sulfhydration and sulfonation, which are thought to control parkin activity, its subcellular localization, conformation, and solubility^24,31^. Decreased parkin solubility and formation of high-molecular-weight (HMW) parkin aggregates are reported with missense mutations or dopamine exposure, using in vitro experiments or in *post-mortem* tissues^32–36^. More particularly, a recent study showed an association between a gradual increase in parkin oxidation and insolubility with age in multiple human brain regions, including the SN^37^. Such a loss of function of parkin could impair the ubiquitin-proteasome system, leading to oxidative damage and mitochondrial dysfunction, which is known in PD to contribute to degeneration of dopaminergic neurons^24,38–41^. In brief, while there is strong evidence for a role of parkin in various vital cell survival pathways, whether a parkin dysfunction contributes to idiopathic PD cases remains speculative.

Relatively few clinicopathological studies have focused on the SN, despite the critical importance of this region in PD. Here, we took advantage of the Saskatchewan brain deposit on movement disorders providing detailed clinical patient characterization to investigate parkin and αsyn changes in terms of solubility and concentrations in multiple brain regions of idiopathic PD patients (*n* = 24) and controls individuals (*n* = 21), in relation with dopamine loss, disease duration and response to levodopa. This is the first demonstration that high molecular weight parkin aggregates accumulate in the SN of idiopathic PD patients.

## Results

### Higher post-mortem levels of insoluble high molecular weight (HMW) parkin in the SN of PD patients

**Fig. 1:**
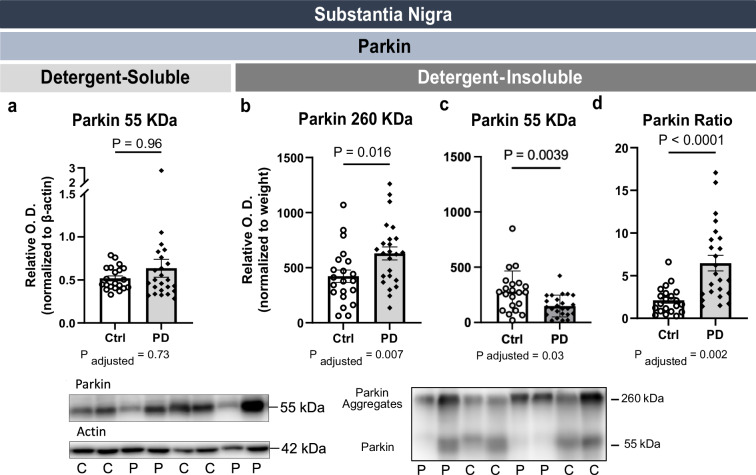
Higher HMW and lower monomers of insoluble parkin in the substantia nigra of PD patients. While detergent-soluble native parkin (55 kDa) levels were unchanged (**a**), higher levels of detergent-insoluble HMW parkin (260 kDa) (**b**) and lower levels of detergent-insoluble native parkin (55 kDa) (**c**) were detected in the SN of PD patients compared to controls. This leads to a higher detergent-insoluble parkin (260/55) ratio in the SN of the PD group compared to the control group (**d**). Statistical analysis: data are represented as mean ± SEM (*N* = 21 ctrl and 24 PD); *P*-value from Mann-Whitney tests are shown on top, while *P*-value after adjustments for age and sex are provided below the graphs. Data in (**b**, **c**) were normalized with SN sample weight (mg). Ctrl/C control individuals, HMW high molecular weight, PD/P Parkinson’s disease patients, SEM standard error of the mean, SN substantia nigra, O.D. Optical Density. Representative WB of consecutive bands were shown from the samples. Full images are shown in Supplementary Fig. 1.

Despite the genetic link between parkin and PD, *post-mortem* parkin levels in the SN of idiopathic PD patients have not been investigated^42^. In SN homogenate fractions containing soluble proteins, we observed a band corresponding to parkin migrating at its expected molecular weight (55 kDa), with average levels being comparable between PD patients and controls (Fig. 1a). Full images of Western blots (WB) are shown in Supplementary Fig. 1. However, in fractions of insoluble proteins, beside the monomeric full-length parkin (55 kDa), we also detected an immunosignal at approximately 260 kDa, corresponding to a high molecular weight (HMW) form of parkin, akin to previous observations^32,35,37,43^. Interestingly, levels of this apparently aggregated form of parkin were higher in PD patients (+49%) along with lower levels of insoluble monomeric parkin (−47%) (Fig. 1b, c), translating into a ratio of HMW/monomeric parkin that is twice higher in the SN of PD patients (Fig. 1d). Adjustments made for age and sex did not change the statistical significance of the comparisons. These observations were further corroborated using a polyclonal antibody raised against the C-terminus of parkin in additional Western blot analyses (Supplementary Fig. 2).

### Higher post-mortem levels of αsynP129 in the SN of PD patients

**Fig. 2:**
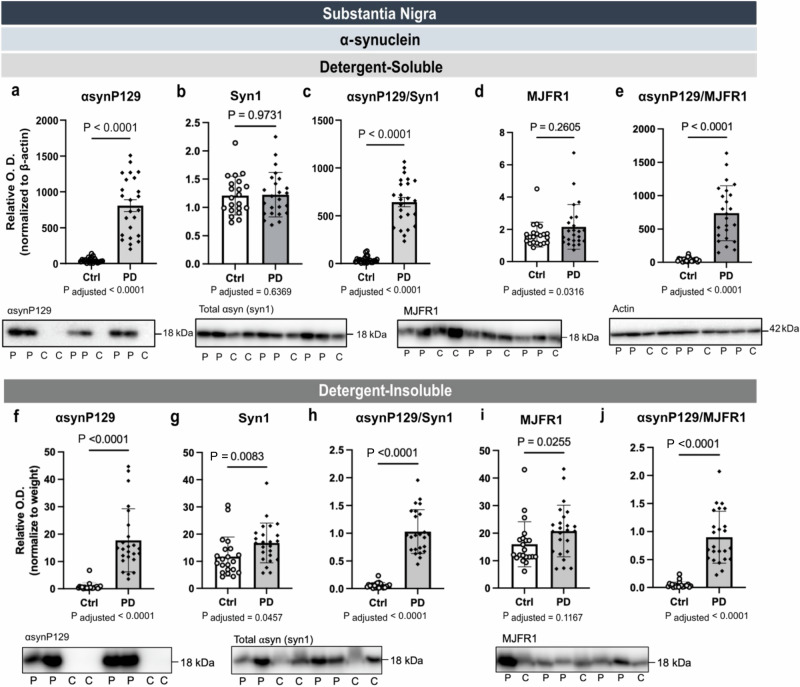
Higher post-mortem levels of αsynP129 in the substantia nigra of PD patients. Levels of αsynP129 were higher in detergent-soluble and detergent-insoluble fractions extracted from the SN of PD patients (**a**, **f**). Differences in total αsyn levels when measured with SYN1 and MJFR1 antibody were overall nonsignificant between groups in detergent-soluble (**b**, **d**) detergent-insoluble fractions (**g**, **i**). Accordingly, the αsynP129/total αsyn ratios were also higher in PD in both fractions (**c**, **e**, **h**, **j**). Statistical analysis: data are represented as mean ± SEM (*N* = 21 ctrl and 24 PD); *P*-value from Mann-Whitney tests are shown on top, while *P*-value after adjustments for age and sex are provided below the graphs. The normalization of O.D. values in the detergent-insoluble fraction was done over the SN weight (mg). Ctrl/C control individuals, PD/P Parkinson’s disease patients, SEM standard error of the mean, SN substantia nigra, αsyn α-synuclein, αsynP129 α-synuclein phosphorylated at serine 129, O.D. Optical Density. Representative WB of consecutive bands were shown from the samples. Full images are shown in Supplementary Fig. 3.

Relatively few studies have documented *post-mortem* changes of αsyn in the SN of idiopathic PD patients and most have used qualitative immunofluorescence and immunohistochemistry^42,44–46^. Although αsynP129 is considered to be the most predominant αsyn PTM^11–14,20^, few studies have sought to quantify it in the human SN. Therefore, we determined the levels of both αsynP129 and total αsyn in SN homogenates using WB. Full images of WB are shown in Supplementary Fig. 3. In soluble fractions, the levels of αsynP129 were ~17 times higher in PD patients compared to controls (Fig. 2a), whereas no significant differences were observed for total αsyn levels (Fig. 2b, d). The ratio of αsynP129 over total αsyn (immunodetected using SYN1 or MJFR1 antibodies) was accordingly ~15 times higher in PD patients compared to controls (Fig. 2c, e). In formic acid extracts containing detergent-insoluble proteins, average levels of αsynP129 were 22 times higher in PD patients compared to controls (Fig. 2f). Total αsyn levels were slightly higher in PD, losing significance after adjustment for age and sex (Fig. 2g, i). The corresponding αsyn phosphorylation ratios were on average ~18 or ~19 times higher in PD patients, respectively (Fig. 2h, j). Adjustments made for age and sex did not change the strong statistical significance for comparisons of αsynP129 levels between groups.

### Changes in insoluble parkin in PD is restricted to the SN, whereas changes in phosphorylated αsyn are more widespread

**Fig. 3:**
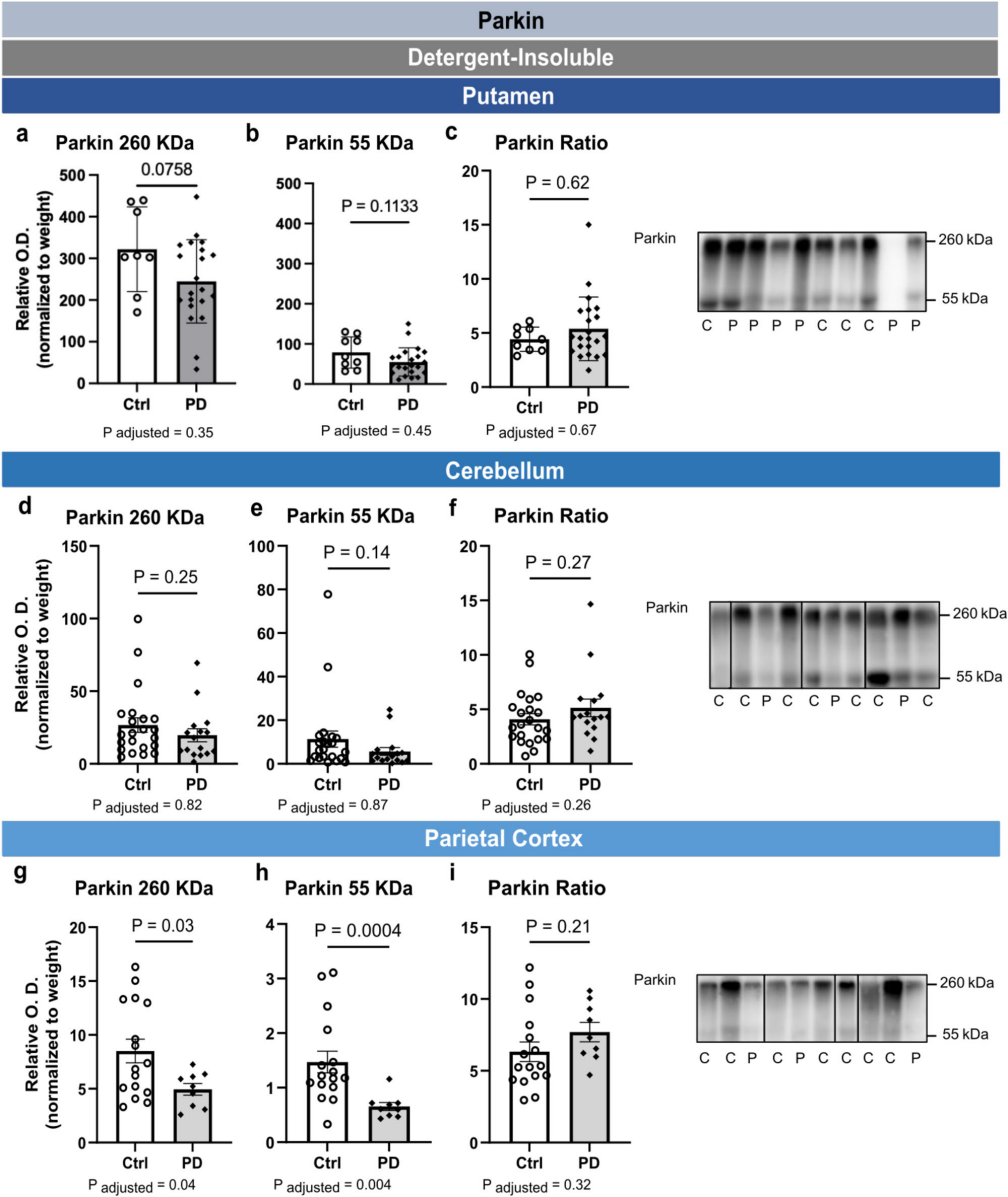
Post-mortem levels of detergent-insoluble parkin in putamen, cerebellum and parietal cortex of PD patients. No differences were detected between controls and PD patients for parkin aggregates (260 kDa), native parkin (55 kDa) and the parkin ratio (260/55) both in the putamen (**a–c**) and the cerebellum (**d–f**). In the parietal cortex, although PD patients had lower levels of parkin HMW species (**g**) and parkin native proteins (**h**), the differences in the parkin ratio levels remained nonsignificant (**i**). Statistical analysis: data are represented as mean ± SEM (Putamen, *N* = 9 ctrl and 22 PD; Cerebellum, *N* = 22 ctrl and 16 PD; Parietal Cortex, *N* = 16 ctrl and 9 PD); unpaired Student T test (**a**, **g**, **i**); Mann-Whitney test (**b–f**, **h**); the *P*-value after adjustments for age and sex is provided; All O.D. values from detergent-insoluble fractions were normalized over sample weight (mg). Ctrl/C control individuals, PD/P Parkinson’s disease patients, HMW high molecular weight, SEM standard error of the mean, O.D. Optical Density. Representative WB of bands were shown from the samples, where the inserted black vertical line indicates nonconsecutive bands. Full images are shown in Supplementary Fig. 6.

Idiopathic PD is characterized by a remarkable vulnerability of dopaminergic neurons in the SNpc^47,48^. To investigate the regional specificity of parkin changes noted in the SN, similar experiments were performed in the putamen, cerebellum and parietal cortex. Full images of WB are shown in Supplementary Fig. 4. In insoluble fractions from putamen, cerebellum and parietal cortex, both HMW and monomeric parkin could be detected. However, no significant differences between groups were observed (Fig. 3a–f), except that both forms of parkin were less present in the PD group compared to the control group in the parietal cortex (Fig. 3g, h). We also analyzed parkin immunosignal in tris-buffered saline (TBS) and detergent-soluble fractions from these three brain regions and no differences were found between groups (Supplementary Figs. 5, 7 along with full images of WB shown in Supplementary Figs. 6, 8). Adjustments for age and sex did not change the statistical significance of these comparisons. Taken together, these data suggest that the conversion of monomeric parkin into an insoluble HMW form is a molecular outcome restricted to the SN in PD.

**Fig. 4:**
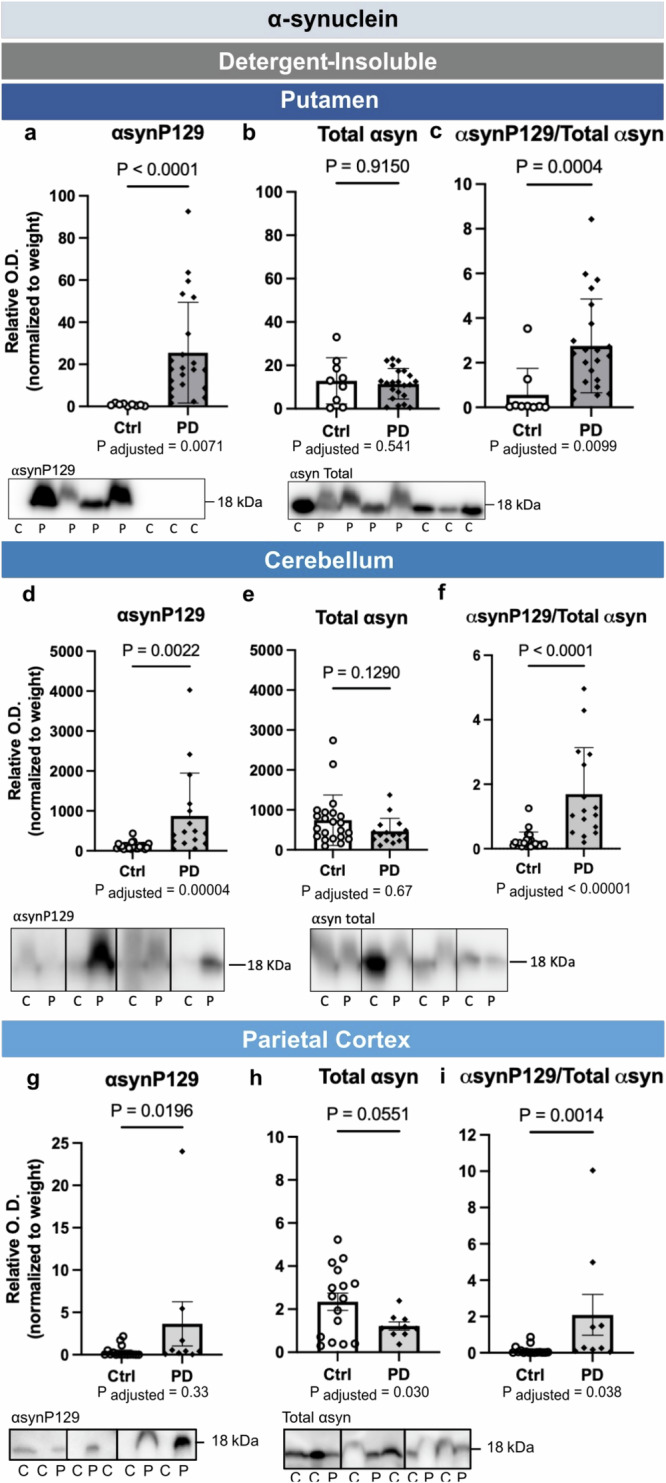
Post-mortem levels of detergent-insoluble αsyn in putamen, cerebellum and parietal cortex of PD patients. In putamen, cerebellum and parietal cortex, PD patients had higher levels of αsynP129 (**a**, **d**, **g**) and αsynP129/total αsyn ratio (**c**, **f**, **i**), with or without adjustments for sex and age (except in the cortex where significance of αsynP129 was lost after adjustment). By contrast, no significant differences in total αsyn (**b**, **e**, **h**) levels were noted between groups in these regions. Statistical analysis: data are represented as mean ± SEM (Putamen, *N* = 9 ctrl and 22 PD; Cerebellum, *N* = 22 ctrl and 16 PD; Parietal Cortex, *N* = 16 ctrl and 9 PD); *P*-values shown are from Mann-Whitney (top) and analysis of covariance with age and sex as covariates (bottom); All O.D. values from detergent-insoluble fractions were normalized over sample weight (mg). Ctrl/C controls, PD/P Parkinson’s disease patients, αsyn α-synuclein, αsynP129 α-synuclein phosphorylated at serine 129, SEM standard error of the mean, O.D. Optical Density. Representative WB of bands are shown, where the inserted black vertical line indicates nonconsecutive bands. Full images are in Supplementary Fig. 9.

Next, using the same experimental approach, we assessed changes in αsyn levels in the putamen, cerebellum and parietal cortex. In formic acid extracts, we first observed that the presence of αsynP129 was not detectable in these regions in a significant proportion of subjects, while total αsyn was more consistently present (Fig. 4a–i). Full membrane images are depicted in Supplementary Fig. 9. When comparing groups, despite large interindividual variability, we did observe statistically significantly higher average levels of αsynP129 and higher αsynP129/total αsyn ratio in PD patients in cerebellum and parietal cortex (Fig. 4d, f, g, i). After adjustments for age and sex, these differences remained significant only for the αsynP129/total αsyn ratio (Fig. 4f, i). As for the total αsyn levels adjusted for age and sex, they were slightly lower in the parietal cortex (Fig. 4h) in PD patients compared to the controls. In summary, despite a statistically significant higher phosphorylation status of αsyn in the cortex and cerebellum, the magnitude of the difference between idiopathic PD and controls was much more clear-cut in the SN than in other brain regions.

### Proteomic confirmation of Parkin in excised Western blot bands

To confirm the identity of parkin in Western blots from both the putamen and the substantia nigra, gel fragments corresponding to the HMW bands were excised and analyzed by LC–MS/MS. Proteomic analysis identified parkin (UniProt A0A669KBE3) with high confidence, based on the detection of five exclusive unique peptides corresponding to 21% sequence coverage (Supplementary Fig. 10). Identified peptides mapped to multiple regions of the parkin protein, supporting specific detection rather than nonspecific band contamination. These results validate the presence of parkin in the Western blot bands used for quantification.

### Post-mortem levels of dopamine and metabolites in the putamen and SN of PD patients

**Fig. 5:**
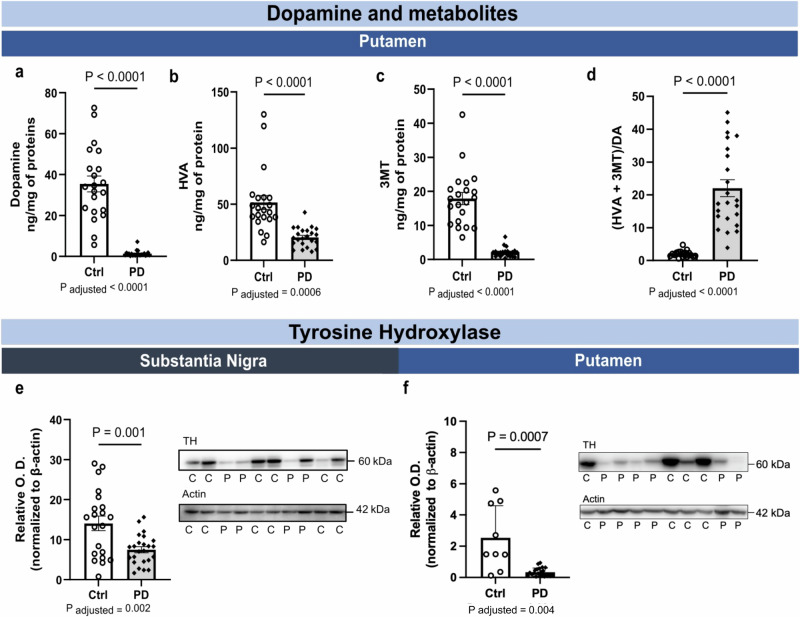
Post-mortem levels of dopamine, metabolites HVA and 3MT and TH in the putamen and in the SN of PD patients. **a** DA concentrations were decreased by over 95% in the putamen of PD patients compared to controls. **b**, **c** Levels of HVA and 3MT, metabolites of DA, were reduced in the putamen of patients with PD, along with a higher (HVA + 3MT)/DA ratio (**d**). TH immunosignal normalized to β-actin in the SN (**e**) and putamen (**f**) were lower in PD patients compared to controls. Statistical analysis: data are represented as mean ± SEM (Putamen, *N* = 9 ctrl and 22 PD; Substantia Nigra, *N* = 21 ctrl and 23 PD); Mann-Whitney test (**a–d**); unpaired student T test (**e**, **f**); the *P*-value after adjustments for age and sex is provided. Ctrl/C control individuals, PD/P Parkinson’s disease patients, SN substantia nigra, DA dopamine, HVA homovanillic acid, 3MT 3-methoxytyramine, TH tyrosine hydroxylase, SEM standard error of the mean, O.D. Optical Density. Representative WB of consecutive bands were shown from the samples. Full images are shown in Supplementary Fig. 11.

The loss of dopaminergic neurons in the SN is the main pathological hallmark of PD^1–4^. Determining the concentrations of dopamine and its metabolites in the putamen remains the most quantitative assessment of nigrostriatal denervation. To that purpose, we utilized high-performance liquid chromatography with electrochemical detection (HPLC-EC). As anticipated, we found that PD patients display a massive reduction in the levels of dopamine (-96%) compared to controls (Fig. 5a). Levels of homovanilic acid (HVA) (Figs. 5b) and 3-methoxytyramine (3MT) (Fig. 5c), which are metabolites of dopamine, were also reduced (−60% and −89% versus Controls), but to a lesser extent than dopamine levels. Consequently, the metabolites/dopamine ratio was higher in PD patients by 10 times compared to controls (Fig. 5d), consistent with an accelerated dopamine turnover in PD patients^1–3^. A more commonly used but less quantitative index of nigrostriatal denervation, the tyrosine hydroxylase (TH) immunosignal was also reduced in both the SN and the putamen in PD patients compared to controls (-47% and -82%, respectively) (Fig. 5e, f). Full membrane images are shown in Supplementary Fig. 11.

### Correlative analyses between parkin, αsyn, catecholamine and LRRK2 levels

**Fig. 6:**
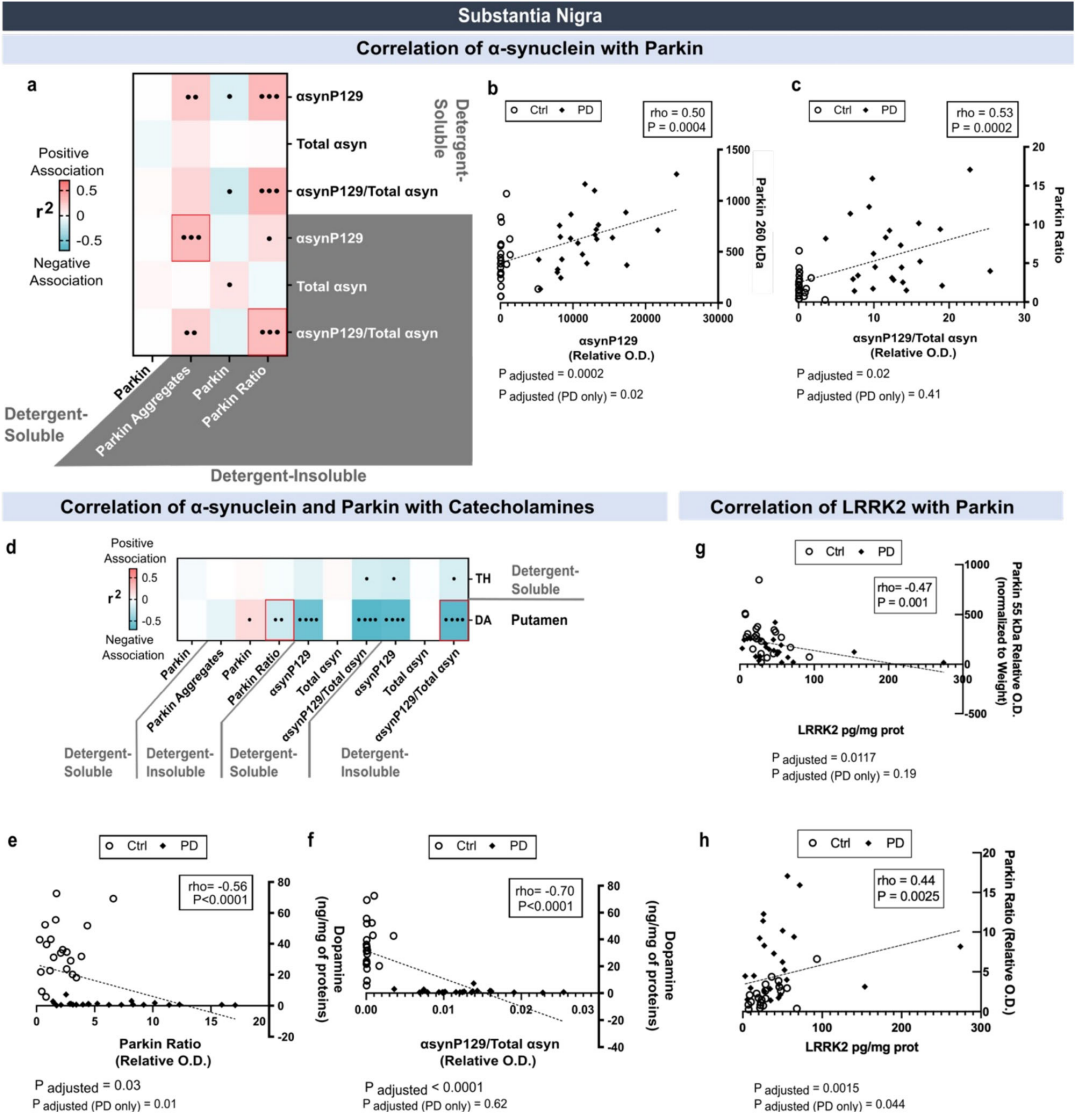
Correlations of *post-mortem* parkin, αsyn in the SN and dopamine or TH in the putamen, and LRRK2 in the SN of PD patients. **a** The correlations of αsyn levels with parkin levels were computed in the SN. Positive correlations between the αsynP129 levels and parkin 260 kDa (**b**) and between the αsynP129/Total αsyn and parkin ratio (**c**) are shown. Correlations of putamen levels TH and DA with SN levels of parkin and αsyn were also analyzed (**d**). Negative correlations between DA levels in the putamen and the SN parkin ratio and (**e**) with the SN ratio of αsynP129/Total αsyn (**f**) are shown. LRRK2 SN levels correlate negatively with soluble parkin (55 kDa) (**g**) and positively with the insoluble parkin ratio (**h**). Statistical analysis: Spearman’s rank correlations were conducted to evaluate associations among the studied variables. Simple linear regressions were performed to generate coefficients of determination (r²). Correlations were adjusted for age and sex to generate *p*-values (•*p* < 0.05; ••*p* < 0.01; •••*p* < 0.001; ••••*p* < 0.0001). Adjusted *p*-values are provided for correlations in the full cohort as well as for correlations calculated within the PD group only. Red and blue cells indicate significant positive and negative correlations, respectively. Red rectangles around cells in (**a**, **d**) correspond to the selected correl**a**tions shown in (**b**, **c**) and (**e**, **f**), respectively. Panels (**b**, **c**, **e**–**h**) underwent the same analyses: linear regression for r², Pearson or Spearman correlation depending on normality, and adjusted correlations for age*sex in the full cohort and in the PD group. Ctrl/C control individuals, PD/P Parkinson’s disease patients, SN substantia nigra, αsyn α-synuclein, αsynP129 α-synuclein phosphorylated at serine 129, DA dopamine, TH tyrosine hydroxylase, O.D. Optical Density.

We then investigated the relationship between parkin and αsyn with nigral denervation. First, we observed a significant positive correlation between the HMW 260-kDa parkin and αsynP129 in insoluble fractions of the SN (Fig. 6a). This association was also significant within PD patients (Fig. 6b, c). By contrast, a weak inverse correlation was detected between monomeric parkin and αsynP129 in insoluble fractions of the SN (Fig. 6a). Accordingly, the parkin HMW/monomers ratio was strongly associated with the accumulation of insoluble αsynP129 /total αsyn ratio (Fig. 6c). No significant association was found for soluble parkin. Second, we observed an inverse association between the parkin aggregation ratio in the SN and dopamine in the putamen (Fig. 6d). Weaker inverse correlations with TH levels of the SN were also noted (Fig. 6d). The significance of the association between the parkin ratio and dopamine levels remained robust when analyzed specifically in PD patients (Fig. 6e). Inverse correlations were also found between dopamine concentrations and soluble as well as insoluble αsynP129 (Fig. 6d, f). No similar association between dopamine levels, αsyn and parkin levels were found in the putamen. Finally, given the importance of LRRK2 activity in PD^49–51^, we proceeded to the analysis of LRRK2 levels by ELISA in the SN and found strong correlations with insoluble 55 kDa parkin (inverse) and 260/55 parkin ratio (Fig. 6g, h). These associations remained significant after adjustment for age and sex, indicating that parkin conversion into a HMW from may be related to elevated LRRK2 levels. In summary, the rise in parkin HMW species in the SN was associated with (i) αsynP129 and αsynP129/total αsyn, the latter only when including PD and controls, (ii) the extent of DAergic denervation, including within PD patients, and (iii) higher LRRK2 levels.

### Experimental nigral degeneration and α-synucleinopathy does not induce parkin aggregation

**Fig. 7:**
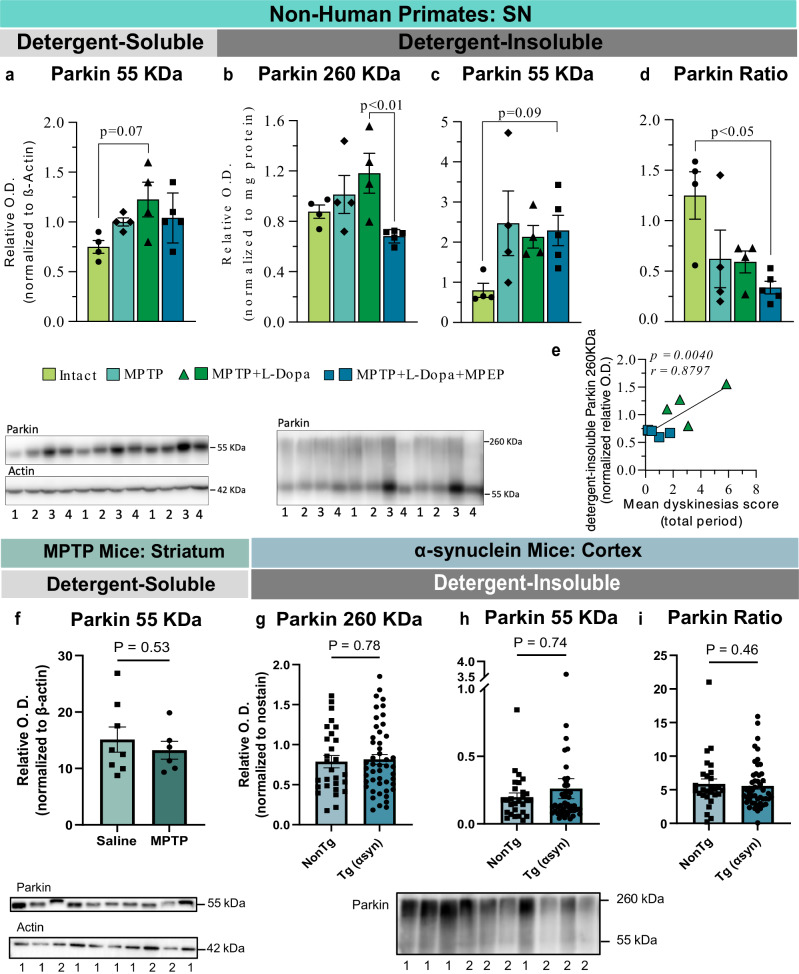
Post-mortem levels of parkin in SN of MPTP-treated non-human primates, in striatum of MPTP mice and in cortex of α-synuclein mice. Native monomeric detergent-soluble parkin (55 kDa) levels appeared higher in the MPTP + L-DOPA group compared to the intact group, without reaching statistical significance (**a**) although in the detergent-soluble fraction it the 55 KDa parkin level are non-significantly lower in the intact group (**c**). MPEP reduced parkin aggregates (260 kDa) in comparison with the MPTP + L-Dopa treated group while the parkin ratio (260/55) decreased compared to the intact group (**b**, **d**). **e**. Positive correlation between detergent-insoluble parkin 260 KDa and dyskinesia score. In MPTP-denervated mice, the differences in native parkin levels were nonsignificant (**f**) in the detergent-soluble fraction of the striatum In αsyn transgenic mice, no differences were detected between the NonTg and Tg (αsyn) groups for parkin aggregates (260 kDa), monomeric parkin (55 kDa) and the parkin ratio (260/55) levels in the detergent-insoluble fraction of the cortex (**g–i**). Statistical analysis: data are represented as mean ± SEM (MPTP monkeys: *N* = 4 intact group, 4 MPTP group, 5 MPTP + L-DOPA group, 5 MPTP + L-DOPA + MPEP group; αsyn mice: *N* = 28–31 NonTg and 48 Tg (αsyn); MPTP mice: *N* = 8 saline and 6 MPTP); Kruskal-Wallis test followed by Dunn’s multiple comparisons test (**a–d**); unpaired student T test (**f**, **g**); Mann-Whitney test (**h**, **i**); the normalization of O.D. values in the detergent-soluble fraction was done over β-actin while the normalization in the detergent-insoluble fraction was done over mg of total protein (primates) and Nostain values (mice). Legend for MPTP monkeys WB: 1, intact; 2, MPTP; 3, MPTP + DOPA; 4, MPTP + L-DOPA + MPEP. Legend for αsyn mice WB: 1, NonTg; 2, Tg (αsyn). Legend for MPTP mice WB: 1, saline; 2, MPTP. MPTP 1-methyl-4-phenyl-1,2,3,6-tetrahydropyridine, L-DOPA levodopa, MPEP 2-methyl-6, SN substantia nigra, αsyn, α-synuclein, NonTg nontransgenic, Tg transgenic, SEM standard error of the mean, O.D. Optical Density. Representative WB of bands were shown from the samples, where the inserted black vertical line indicates nonconsecutive bands. Full images are shown in Supplementary Figs. 12, 13.

Observations from *post-mortem* human PD samples do not conclusively reveal causal relationships, as these samples are collected significantly after the onset of pathological events. To get further insight into whether such an apparent aggregation of parkin is a consequence of dopaminergic neuronal loss, we measured parkin levels in mice and non-human primates exposed to the neurotoxin MPTP. Samples used here were from previous published studies in which MPTP non-human primates and mice had a 98% and 78% reduction of dopamine in the putamen, respectively^52,53^. We observed that the MPTP insult in non-human primates had no significant impact on soluble and insoluble monomeric parkin (55 kDa) or HMW parkin (Fig. 7a–c). Contrasting with what we found in idiopathic PD, there was instead a trend toward higher monomeric parkin in MPTP-treated animals (Fig. 7c). Five MPTP animals were treated with the mGlu5 receptor negative allosteric modulator (NAM) 2-methyl-6-(phenylethynyl) pyridine (MPEP) which was shown to reduce the development of levodopa-induced dyskinesias^54^. These animals displayed lower HMW parkin and 260/55 ratio (Fig. 7b–d). A significant correlation between HMW parkin and dyskinesias score was observed (Fig. 7g). No changes were detected in striata harvested from mice following the MPTP insult (Fig. 7e). Full membrane images are shown in Supplementary Figs. 12, 13. This suggests that parkin aggregation cannot be replicated by simply generating dopaminergic neuronal loss.

Finally, detergent-insoluble parkin was also investigated in cortices collected from an animal model of α-synucleinopathy (Thy1-αSyn mouse)^55^, and no differences were found between groups (Fig. 7f–h), suggesting that parkin aggregation is not a consequence of widespread synucleinopathy.

### Relationship between SN levels of HMW parkin and αsyn and clinical characteristics of the PD patients

**Fig. 8:**
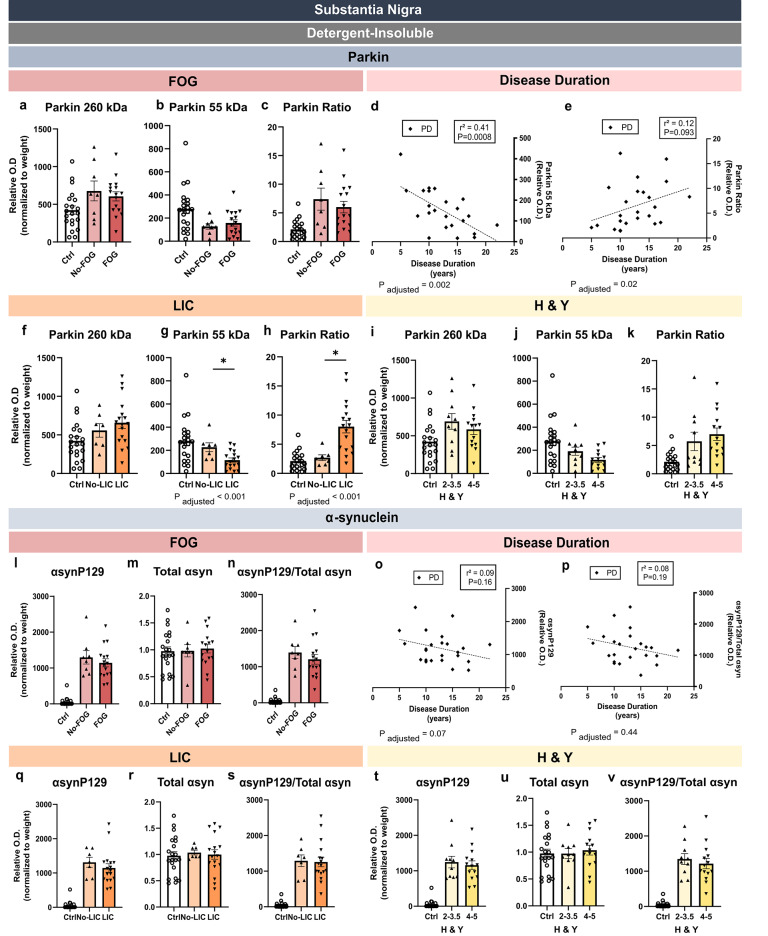
Detergent-insoluble levels of parkin and αsyn in SN in relation to clinical data. Detergent-insoluble parkin (260 kDa), native proteins (55 kDa) and parkin ratio (260/55) are shown in relation to FOG (**a–c**), to LIC (**g–i**) and to H & Y (**j–l**). The correlations of parkin and parkin ratio levels of PD patients with disease duration were computed (**d**, **e**). Briefly, when the PD patients were separated into two groups according to the presence of FOG, the presence of LIC or the stages on the H & Y scale, no differences were observed between the two PD groups, except for the LIC patients that had higher parkin ratio levels. In addition, parkin ratio levels positively correlated with disease duration, after adjusting for sex and age. Detergent-insoluble αsynP129, total αsyn and the αsynP129 over the total αsyn ratio are shown in relation to FOG (**l–n**), to LIC (**q–s**) and to H & Y higher (**t–v**). The correlations of αsynP129 and αsyn ratio levels of PD patients with disease duration were also computed (**o**, **p**). Statistical analysis: data are represented as mean ± SEM (*N* = 21 ctrl and 24 PD); one-way ANOVA followed by Tukey’s or Dunnett’s post-hoc tests (**a**, **f**, **i**, **m**, **r**, **u**); Kruskal-Wallis test followed by Dunn’s multiple comparisons test (**b**, **c**, **g-h**, **j–l**, **n, q**, **s**, **t**, **v**); *p < 0.05; simple linear regressions were performed to generate coefficients of determination (r^2^); correlations were adjusted for age and sex to generate p-values. The adjusted p-value according to sex and age is provided; the normalization of O.D. values in the detergent-insoluble fraction was done over the SN weight (mg). Ctrl control individuals, PD Parkinson’s disease patients, SN substantia nigra, αsyn α-synuclein, αsynP129 α-synuclein phosphorylated at serine 129, O.D. Optical Density, FOG freezing of gait, LIC levodopa-induced complications, H & Y Hoehn & Yahr Scale, SEM standard error of the mean, ns nonsignificant.

PD is a heterogenous disease with a wide clinical spectrum^8,56,57^. Taking advantage of the available clinical data, we first found no difference in insoluble parkin levels between PD patients with or without freezing of gait (FOG) (Fig. 8a–c). When the levels of insoluble monomeric (55 kDa) parkin were examined in relation to disease duration, which lasted 13 years on average, a significant negative correlation was observed (Fig. 8d). The parkin 260/55 kDa ratio positively correlated with disease duration, but only after adjusting for sex and age (Fig. 8e). While parkin levels did not statistically differentiate PD patients with levodopa-induced complications (LIC) and those without, only PD subjects with LIC displayed a higher parkin 260/55 ratio than controls (Fig. 8f–h). After separating according to the stages on Hoehn & Yahr Scale (H & Y), differences between parkin levels of the 2–3.5 score PD group and the 4–5 score PD group were nonsignificant (Fig. 8i–k). By contrast, no significant association between SN levels of αsyn and FOG, disease duration, LIC and H & Y stages was detected (Fig. 8l–v), suggesting that the increase in αsynP129 levels is a general characteristic of all patients with a PD diagnosis. Overall, these results indicate that the progression of PD is accompanied by a reduction in the monomeric form of parkin, which is converted into a HMW insoluble form, more prominently in individuals experiencing LIC.

## Discussion

We investigated the relative concentrations and changes in solubility of parkin and αsyn in multiple brain regions of idiopathic PD patients (*n* = 24) followed in the Saskatchewan Movement Disorders Program and control individuals (*n* = 21). In summary, in individuals with a PD diagnosis, we observed: (i) higher levels in the SN of insoluble HMW parkin migrating at 260 kDa, along with lower levels of 55-kDa monomers; (ii) higher levels of αsynP129 in the SN and other brain areas; (iii) a loss of dopamine in the putamen, which was inversely associated with HMW parkin and αsynP129 in the SN; and (iv) higher ratios of HMW/monomeric parkin in subjects with LIC and longer disease duration. This study adds parkin conversion into a HMW form in the SN as a novel pathognomonic sign of idiopathic PD, alongside with phosphorylation of α-synuclein at serine-129 in the SN and dopamine loss in the putamen.

The main observation reported in the present manuscript is the higher *post-mortem* level of insoluble parkin migrating at 260 kDa combined with reduced monomers of parkin in the SN from PD patients. This observation, confirmed using two different antibodies, suggests that a conversion of parkin into a HMW form occurs progressively in PD specifically in the SN. Importantly, the presence of parkin in this HMW band in the insoluble fractions was confirmed using LC–MS/MS analysis of excised gel bands. The concept of decreased parkin solubility has been previously suggested as a mechanism of loss of function following mutations^32–37^. The parkin protein can undergo various post-transcriptional modifications (such as oxidation, S-nitrosylation, sulfonation and catecholation) affecting its structure and solubility, which could explain the HMW parkin species observed here^32,35–37,43,58,59^. Three of these publications hinted toward higher insoluble monomeric parkin in the caudate/putamen in a smaller number sample from PD brains compared to controls, but levels in the SN were not examined^37,43,59^. A key *post-mortem* study reported that parkin solubility declined with age in the human brain, including the SN, in association with oxidative damage^37^. We observed no association with age, but this is likely due to the narrow range of ages in the individuals studied. More importantly, however, we did observe an association between a parkin conversion and longer disease duration, which suggests a key involvement of parkin in the PD neurodegeneration process. In sum, the present results offer the first evidence of the conversion of parkin into an insoluble HMW form as a pathophysiological event occurring in the SN of idiopathic PD patients.

Parkin conversion into an insoluble HMW form in the SN was associated with dopamine loss. Whether these pathological events occurred before, after or parallel to dopamine loss is unknown. However, the absence of significant changes in parkin following a massive nigrostriatal denervation in animals strongly suggests that the observed changes in parkin are not a mere consequence of dopamine loss. On the other hand, there is ample evidence in the literature that a loss of parkin function can affect dopamine cells. First, a loss of parkin activity is a key consequence of mutations in the gene coding for parkin, known to cause juvenile autosomal recessive PD^24–26^. TH-positive neurons derived from induced pluripotent stem cells from PD patients with a parkin mutation also show defects in dopaminergic neurotransmission and toxicity^60^. A wealth of data from several experimental paradigms indicate that a loss of parkin enzymatic activity impairs proteasomal degradation of substrates, leading to defective mitochondria and oxidative damage, contributing to neurodegeneration^24,38–40,61^. Given the association observed here between parkin aggregation and higher LRRK2 levels, it becomes interesting to note that these two proteins are known to physically interact, and that enhanced LRRK2 activity was shown to reduce the mitophagic function of parkin in vitro^49,51,62^. Based on this, it could be hypothesized that the conversion of parkin into a HMW defective form could result in a loss of function contributing to the death of dopaminergic neurons in the SN. In sum, although the pathological role of sequestrating parkin into insoluble HMW forms remains to be elucidated, there are plausible mechanisms by which it can alter DAergic cell function and vulnerability in PD.

For several decades, the αsyn protein has been one of the most extensively studied proteins in PD. This interest stems from its established role as a key component of Lewy bodies and genetic evidence linking mutations and gene triplications with parkinsonism^5,8–10,15,44–46,63^. Here, we found that relative levels of soluble and insoluble αsyn phosphorylated at serine 129 were dramatically increased in PD patients compared to controls in the SN as well as in other brain regions. By contrast, total αSyn levels in the SN, cerebellum, putamen, and cortex did not clearly distinguish PD patients from controls. This implies that while formation of HMW parkin affects specifically the SN, the αsynP129-associated proteinopathy at least partly extends to other brain regions. These observations also suggest that therapeutic interventions simply aiming at decreasing the total amount of αsyn might not be sufficient without specifically targeting its hyperphosphorylation, as proposed previously^9,16–18,64^.

The accumulation of insoluble parkin species migrating at 260 kDa showed a significant association with levels of insoluble αSynP129 in the SN. This positive correlation may stem from impaired ubiquitin-proteasome system function, caused by reduced parkin activity, which would compromise the clearance of α-synuclein^30,32,65^. Notably, co-expression of parkin with α-synuclein in rats has been shown to promote PTMs of α-synuclein, particularly its phosphorylation at serine 129^66^. Interestingly, our findings demonstrate that inducing synucleinopathy in mice does not lead to increased HMW parkin. This suggests that parkin dysfunction likely occurs in parallel or exert a causal role in the pathological mechanisms contributing to synucleinopathy and PD. Another notable observation was that HMW parkin rises as the disease progresses, whereas αsynP129 does not, indicating some divergence between the two pathological processes. In sum, the results of the present study, which analyzed both parkin and α-synuclein within the same sample series, point toward a possible link between these two proteinopathies and their specific detrimental effect on the dopaminergic system.

Among PD patients, the ratio increase in HMW/monomeric parkin was significant only in those who developed motor complications following levodopa treatment (LIC). A possible explanation is that levodopa treatment could have triggered parkin aggregation in the SN, resulting in motor complications^67,68^. However, levodopa treatment of MPTP monkeys did induce dyskinesias, without leading to the formation of insoluble HMW parkin. Nevertheless, the anti-dyskinetic compound MPEP reduced the 260/55 ratio, suggesting that the absence of LIC in denervated animals is associated with less conversion to HMW parkin. Several other common factors present in patients with LIC and parkin aggregates may also explain this association, including longer treatment and/or disease duration, as well as more extensive DAergic denervation^69,70^. It remains interesting to consider that HMW parkin aggregates in PD patients could be a predictor for LIC.

Although the described relative accumulation of parkin into a HMW species clearly distinguishes patients from controls, and parkin presence was confirmed using LC/MS-MS, its exact structure is not well characterized. Additional techniques such as Fourier-transform infrared spectroscopy or circular dichroism spectroscopy would be necessary to assess secondary structure of parkin found in HMW forms, such as α-helical structures or those with β-pleated sheets^71^. Finally, several limitations inherent to human post-mortem studies should be acknowledged. Human brain samples display substantial inter-individual variability, and there is no universally optimal method for data normalization, particularly when working with insoluble fractions containing abnormally aggregated proteins. Moreover, due to neuronal loss, SN samples from individuals with OD yielded less tissue than control samples, which may introduce additional variability. Therefore, these findings would benefit from replication in an independent cohort.

Overall, this study brings new insights on the implication of proteinopathies involving parkin and αsyn in idiopathic PD, possibly establishing parkin aggregation in the SN as a new pathognomonic marker for idiopathic PD, beyond α-synuclein phosphorylation at serine-129 and the loss of dopamine in the putamen. The association observed with the duration of the disease and the absence of similar changes in acute models of nigral denervation indicate that this modification of parkin is not a mere consequence, but an active player in the disease process. While the molecular characterization of HMW parkin remains to be performed, previous work shows that the expected loss of parkin function can contribute to mitochondrial dysfunction and nigral dopaminergic neurodegeneration. In addition to dopamine replacement and treatments targeting the reduction of α-synuclein phosphorylation at serine 129, blocking the conversion of parkin into its HMW form represents a potentially promising therapy for PD.

## Methods

### Human Samples: Saskatchewan brain deposit on movement disorders

Brain samples were obtained from the Saskatchewan Movement Disorders Program (SMDP) where clinical data collected from patients enabled longitudinal follow-up^56,72^. Consent for brain autopsy and use of brain tissue for research was approved by the University of Saskatchewan Ethics Board as well as the Research Ethics Committee of the CHU de Québec-Université Laval (018-3985). All procedures involving human participants were conducted in accordance with the ethical standards of the institutional research committees and with the Declaration of Helsinki. Two movement disorder neurologists performed all clinical diagnosis while post-mortem diagnosis was done by a certified neuropathologist. Data such as sex, age at onset, duration of disease, disease severity (Unified Parkinson’s Disease Rating Scale (UPDRS) and modified H & Y scale), prescribed drugs and their adverse effects (including motor complications) were collected. More specifically, disease duration (5–11 years: *n* = 11, 13–22 years: *n* = 13), motor complications (No-LIC: *n* = 7; LIC: *n* = 17) including dyskinesia and wearing off and were recorded during each assessment^56,73^. Freezing of gait (No-FOG: *n* = 8; FOG: *n* = 16) was also documented. The Hoehn & Yahr (H & Y) scale, being the most widely used and accepted staging system, was initially used in order to measure the global severity, followed by the use of the Unified Parkinson’s Disease Rating Scale (UPDRS) and modified H & Y scales (stages 2–3.5: *n* = 10; stages 4–5: *n* = 14)^56,73,74^. Since recruitment was done on a voluntary basis, the controls were slightly younger than the patients, while among the latter, the proportion of men was higher, similar to what is observed in the general population^75^. The cohort characteristics are shown in Supplementary Table 1.

### Autopsy and handling of the brain material

After autopsies were performed within 24 h after death, the collected brains were separated into two: one half of the brain, which was fixed in formalin, was used for histologic and diagnostic studies of the midbrain while the other half was frozen at -80°C and cut along the frontal plane in order to obtain 2–3 mm thick slices. Clinical evaluations were accomplished by one of two movement disorders neurologists (AHR, AR), and the post-mortem diagnosis was done for each patient by a Canadian certified neuropathologist^56,72,73^. Slices corresponding to the parietal cortex, the putamen and the SN (coronal plane) and the cerebellar cortex (axial plane) were used to extract tissues for these regions ($\sim$100 mg). Coronal slices containing the SN were cryostat-sectioned (20 µm), thaw-mounted onto SuperFrostPlus slides (75 × 50 mm), desiccated overnight at 4 °C, and stored at −80 °C until assayed, as described in ref. ^76^. In addition, the SN were dissected on cryostat sections and 5 × 50 µm sections were harvested to obtain approximately 30 mg of frozen sample and stored at −80 °C. Tissue pH was measured as an indication of tissue quality ^76,77^.

### Tissue processing

For Western Blotting experiments, proteins were extracted from tissue homogenates by sequential fractionation using buffers, detergents, and acid, as shown^76,78,79^. The TBS-soluble fraction contains mostly cytosolic and extracellular proteins, the detergent-soluble fraction includes most membrane-bound proteins, and the detergent-insoluble fraction corresponds to insoluble proteins, such as aggregated forms. For the SN, since the available starting material was limited, only two fractionation steps were performed, resulting in a detergent-soluble fraction that also contained TBS-soluble proteins, and a detergent-insoluble fraction. Briefly, a homogenization in TBS buffer (50 mM tris-HCl, 138 mM NaCl, 2.7 mM KCl, with protease and phosphatase inhibitors and 0.1 mM EDTA) was first performed, and samples were sonicated and centrifuged (20 min; 100,000 g) to generate a supernatant, which is the TBS-soluble fraction. Secondly, the resulting pellet was subjected to homogenization in a lysis buffer containing detergents (0.5% of deoxycholate, 150 mM NaCl, 1% of Triton X-100, 10 mM NaH_2_PO_4_, 0.5% sodium dodecyl sulfate (SDS) with protease and phosphatase inhibitors and 0.1 mM EDTA), followed by sonication and centrifugation to generate a supernatant corresponding to the detergent-soluble fraction. Thirdly, the resulting pellet was resuspended in formic acid 99% (100 μl; Sigma-Adrich Cat# F0507), sonicated and centrifuged to produce the detergent-insoluble fraction (formic acid-soluble fraction containing insoluble proteins). The generated supernatant was then evaporated under a fume hood before solubilization in Laemmli buffer (60 mM Tris, 10% glycerol, 2% SDS, 0.0025% bromophenol blue, 2.5% β-mercaptoethanol, pH 8.5) and heated at 95 °C for 5 min. Using the bicinchoninic acid (BCA) protein assay kit (Thermo Fisher Scientific), the protein quantity in the TBS-soluble and detergent-soluble fractions was determined.

### Catecholamine analysis

Levels of dopamine, homovanillic acid (HVA) and 3-methoxytyramine (3MT) were determined by HPLC with electrochemical detection, as described previously^1,53,80^. Cold HClO_4_ (0.1 N) was added to putamen sections, which were then homogenized and centrifuged for 10 minutes at 4 °C (16,000 g). Supernatants were collected and stored at −80 °C while protein pellets were resuspended and incubated in TBS buffer 2X overnight at 4 °C. The pellets were then vortexed, and the quantity of proteins was determined using the BCA protein assay kit (Thermo Fisher Scientific Cat #23225). Supernatants were subjected to HPLC coupled to electochemical detection (Waters, 717 plus Autosampler automatic injector, 1525 Binary Pump, 2465 Electrochemical Detector, Atlantis dC18 column). Standards of dopamine, HVA and 3-MT were injected in parallel to quantitate samples.

### LRRK2 ELISA

For LRRK2 detection in SN detergent-soluble fraction, Meso Scale Discovery Technology was used (R-PLEX Human LRRK2 Assay Kit, # K1511PR, MSD, USA).

### Western Immunoblotting

Proteins from TBS-soluble and detergent-soluble fractions were added to Laemmli 5X buffer and heated at 95 °C for 5 min (denatured). The same amounts of proteins per sample (12 ug for SN and 15 μg for the remaining), were separated on a sodium dodecyl-sulfate (SDS)-polyacrylamide gel by electrophoresis. The proteins were transferred on polyvinylidene fluoride (PVDF; Cytiva Life Sciences) 0.45 µm membranes. For membrane containing detergent insoluble fraction, total proteins were visualized with a No-Stain™ protein labeling reagent (Invitrogen by Thermo Fisher Scientific) before blocking to use as a loading verification. For αsyn detection, the membranes were fixed with 4% paraformaldehyde pH 7.4 for 30 min before blocking. All membranes were blocked with 3% bovine serum albumin (BSA, BioShop Cat# ALB001) in phosphate-buffered saline (PBS)-Tween 0.1% (PBS, Fisher BioReagents Cat# BP399-20; Tween, Sigma-Aldrich) for 1 h at ambient temperature. As for the immunoblots, the antibodies against parkin C-Terminal region (monoclonal parkin clone [PRK8], 1:1,000 and polyclonal parkin (cell signaling cat#2132, 1:1,000)), TH (Pel-Freez Biologicals Cat# P40101-150, 1:1,000), αsyn (SYN1, BD Biosciences Cat# 610787, 1:1,000), MJFR1 (Abcam Cat# ab138501 [MJFR1], 1:1,000) and αsyn phosphorylated at serine 129 (αsynP129) (Abcam Cat# ab168381 [MJF-R13 (8-8)], 1:1,000; Abcam Cat# ab51253 [EP1536Y] 1:500) were used. As a loading control for the TBS-soluble and detergent-soluble fractions, the antibody against β-actin (Applied Biological Materials Cat# G043) was used at 1:5,000. All incubations were done in Superblock™ blocking buffer in PBS (Thermo Fisher Scientific) containing 0.1% Tween 20 and 0,05% sodium azide. After incubation with a primary antibody, the membranes were washed in PBS-Tween 0.1%, followed by an incubation with a horseradish peroxidase (HRP) anti-mouse (Jackson ImmunoResearch Labs) or anti-rabbit secondary antibody (Jackson ImmunoResearch Labs) at 1:40,000 in PBS containing 0.1% Tween 20 and 1% BSA. The detections were done with Amersham Imager 680 (GE Healthcare Bio-Sciences) following revelation with Luminata (Sigma-Aldrich Millipore), a chemiluminescence HRP substrate. For quantification, bands detected in the soluble fractions were normalized to β-actin bands from the same sample. Normalization in the detergent-insoluble fractions were done over the weight of the appropriate brain region (mg). As protein aggregation in the human brain is abnormal and pathology-dependent, particularly in the SN, the detergent-insoluble fraction exhibits substantial interindividual variability. As a result, neither total protein content nor a specific protein can be used as a reliable normalization reference, since control samples naturally contain less aggregated material and no aggregated housekeeping protein is available for normalization. Thus, normalization to the initial tissue weight represents the most valid approach. This was especially critical for SN samples, which are markedly smaller in PD. Band intensities were determined using the Image Lab software (Bio-Rad). Immunoblots are shown in Supplementary Figs. 1–4, 6, 8, 9, 11–13.

### Identification of parkin in the high molecular weight band by Mass spectrometry

For the proteomics experiments, 40 µL of proteins from the insoluble fraction were separated by SDS–PAGE. Proteins were stained with Coomassie G-250 (Bio-Rad Laboratories, ref. #1610786) for 30 min and subsequently washed in Milli-Q H₂O for 20 min. Bands at 260 kDa and above were excised and stored at 4 °C until enzymatic digestion.

Protein digestion and mass spectrometry analyses were performed by the Proteomics Platform of the CHU de Québec Research Center (Quebec, QC, Canada). Gel bands of interest were cut into small pieces. Proteins were reduced with 10 mM DTT and alkylated with 55 mM iodoacetamide. Trypsin digestion was performed using 126 nM of modified porcine trypsin (Sequencing grade, Promega, Madison, WI) at 37 °C for 18 h. Digestion products were extracted using 1% formic acid, 2% acetonitrile followed by 1% formic acid, 50% acetonitrile. The recovered extracts were pooled, vacuum centrifuge dried, and then resuspended with a solution of 0.1% formic acid. The volumes were adjusted to analyze an amount corresponding to 50% from the total volume.

Samples were analyzed by nanoLC/MSMS using a Dionex UltiMate 3000 nanoRSLC chromatography system (Thermo Fisher Scientific) connected to an Orbitrap Fusion mass spectrometer (Thermo Fisher Scientific, San Jose, CA, USA) equipped with a nanoelectrospray ion source and a FAIMS Pro interface. Peptides were trapped at 20 μl/min in loading solvent (2% ACN, 0.05% TFA) on a 5 mm × 300 μm C18 pepmap cartridge (Thermo Fisher Scientific) during 5 min. Then, the pre-column was switched online with a 50 cm × 75 µm internal diameter separation column (Pepmap Acclaim column, ThermoFisher) and the peptides were eluted with a linear gradient from 5 to 40% solvent B (A: 0.1% FA, B: 80% ACN, 0.1% FA) in 30 minutes, at 300 nL/min (60 min total runtime). Mass spectra were acquired with a data dependent acquisition mode using Thermo XCalibur software version 4.3.73.11 using FAIMS compensation voltage set at −60 V and −40 V split in 2 different experiments. Full scan mass spectra (350–1800m/z) were acquired in the orbitrap using an AGC target of 33%, a maximum injection time of 50 ms and a resolution of 120,000. Internal calibration using lock mass on the m/z 445.12003 siloxane ion was used. Each MS scan was followed by acquisition of fragmentation MSMS spectra of the most intense ions for a total cycle time of 1.5 s (top speed mode). The selected ions were isolated using the quadrupole analyzer with 1.6 m/z windows and fragmented by Higher energy Collision-induced Dissociation (HCD) with 35% of collision energy. The resulting fragments were detected by the linear ion trap in rapid scan rate with an AGC target of 33% and a maximum injection time of 50 ms. Dynamic exclusion of previously fragmented peptides was set for a period of 30 s and a tolerance of 10 ppm.

MGF peak list files were created using Proteome Discoverer 2.4 software (Thermo). MGF files were then analyzed using Mascot (Matrix Science, London, UK; version 2.5.1). Mascot was set up to search a contaminant database and a Uniprot Homo sapiens database (83399 sequences, UP000005640 downloaded on 2025.02) database assuming the digestion enzyme trypsin. Mascot was searched with a fragment ion mass tolerance of 0.60 Da and a parent ion tolerance of 10.0 PPM. Carbamidomethyl of cysteine was specified in Mascot as a fixed modification. Deamidation of asparagine and glutamine and oxidation of methionine were specified in Mascot as variable modifications. 2 missed cleavages were allowed.

Scaffold (version Scaffold_5.2, Proteome Software Inc., Portland, OR) was used to validate MS/MS-based peptide and protein identifications. A false discovery rate of 1% was used for peptide and protein. Proteins that contained similar peptides and could not be differentiated based on MS/MS analysis alone were grouped to satisfy the principles of parsimony.

### Animals: MPTP monkeys

Drug-naive ovariectomized female cynomolgus monkeys (*macaca fascicularis*) were continuously injected with MPTP in order to induce a stable Parkinsonian syndrome, which was followed by a levodopa/benserazide treatment while four intact monkeys were used as controls, as previously described^52,54^. The animal study protocol was approved by the animal Research Committee of the CHU de Québec-Université Laval Research Center (2010062). The SN was dissected from frozen section as detailed elsewhere^52^. The detergent-soluble and the detergent-insoluble fractions were obtained following the method described above. At the end of the experiments, all monkeys (*n* = 4 intact controls, 4 MPTP, 5 MPTP + L-DOPA, and 5 MPTP + L-DOPA + MPEP) were euthanized by an overdose of sodium pentobarbital. The time interval between the last drug administration and euthanasia was 24 h. Brains were rapidly removed, immersed in isopentane for <30 s (−40 °C), and stored at −80 °C until use. These samples were obtained from a previously published cohort^52,54^, in which euthanasia procedures were originally described.

### Animals: αsyn mice

Male Thy1-αsyn (transgenic, Tg; *n* = 48) and nontransgenic C57BL/6 (NonTg; *n* = 28–31) mice were bred in our animal research facility from Thy1.2-αsyn mice (line 61) on a full C57BL/6 background, as described previously^55^. Briefly, mice were subjected to a 12:12 h dark:light cycle and were kept in ventilated cages, where one contained between 2 and 5 mice, in addition of having free access to water and fed with different diets (formulated control, no DHA or enriched DHA) not relevant for the present study^55^. Only male mice were used for experimentations because of the transgene’s location on chromosome X and all protocols were approved by the animal Research Committee of the CHU de Québec-Université Laval Research Center (17-110)^55^. At 12 months of age, mice were deeply anesthetized with a ketamine/xylazine mixture (100 and 10 mg/kg, respectively) and euthanized by intracardiac perfusion with phosphate-buffered saline (PBS) containing protease and phosphatase inhibitors. Brains were rapidly collected and hemispheres separated. One hemisphere was snap-frozen and stored at −80 °C for biochemical analyses. These samples were derived from previously published cohorts^55^, in which the full experimental procedures were originally reported.

### Animals: MPTP mice

Male C57BL/6 mice were injected with a MPTP$\cdot$HCl solution (7 i.p injections) freshly dissolved in 0.9% saline at 4 months of age, when the MPTP administration was performed twice on the first two days of the experimental protocol at 12 h-intervals, and once a day on the three subsequent days while the remaining mice were injected with 0.9% saline i.p, as previously described in ref. ^53^. Briefly, mice were kept in groups of 3 to 4 per cage and had free access to food and water. As for the MPTP doses, they were calculated for each mouse with respect to the body surface area. Fourteen days after the last MPTP injection, mice were deeply anesthetized with ketamine/xylazine and euthanized via intracardiac perfusion with PBS containing protease and phosphatase inhibitors. Brains were rapidly collected, the frontal cortex was dissected for fatty acid analyses, and the two hemispheres separated. The right hemisphere was separated at the level of bregma −1.70 mm and the striatum in the rostral section was dissected for Western blotting. These procedures have been previously described in ref. ^53^ and are reiterated here for clarity.

### Statistical analysis

To compare two groups, Mann-Whitney tests were used except in some instances unpaired student’s *t*-test were used when normal distribution and equal variance were assumed. For comparisons involved two or more groups, the parametric one-way ANOVA test as well as the nonparametric Kruskall-Wallis test depending on the normality test result were done. Following the one-way ANOVA test, the Tukey’s multiple comparisons test was done as a post-hoc test while the Kruskall-Wallis test was followed by the Dunn’s multiple comparisons test. Correlative analyses were performed using Pearson or Spearman correlation tests, depending on data distribution. Data were adjusted for age at death and/or sex using distribution and multivariate analyses. A statistical comparison corresponds to a *p*-value lower than 0.05. All statistical analyses were done using GraphPad Prism 9.0 or JMP 16.2.0 software.

## Supplementary information

Supplementary information

## Data Availability

The authors declare that all the data supporting the findings of this study are available within the article and its Supplementary Information files and from the corresponding author on reasonable request.
