# Supplementary material for: High molecular weight insoluble parkin in the substantia nigra of patients with idiopathic Parkinson’s disease
Source: NPJ Parkinsons Dis. 2026 May 7;12:128. doi: 10.1038/s41531-026-01371-2 (PMC13221466; doi:10.1038/s41531-026-01371-2)
Supplement: Supplementary file 1 — Supplementary information [file 41531_2026_1371_MOESM1_ESM.pdf]

Supplementary Information file

Title: High molecular weight insoluble parkin in the substantia nigra of patients with idiopathic Parkinson's disease

Cyntia Tremblay[1], Laura Pshevorskiy[1,2], Rosalie J. Cottez[1,2], Hélèna L. Denis[1], Vincent Emond[1], Marc Morissette[1], Ali H Rajput[3,4], Thérèse Di Paolo[1,2], Alex Rajput[3,4] and

Frédéric Calon[1,2]

1. Axe Neurosciences, Centre de recherche du CHU de Québec – Université Laval, Québec, Québec, Canada

2. Faculté de pharmacie, Université Laval, Québec, Québec, Canada

3. Movement Disorders Program, University of Saskatchewan, Saskatoon, Saskatchewan, Canada, S7N 5A2

4. Faculty of Medicine, University of Saskatchewan, Saskatoon, Saskatchewan, Canada

Corresponding author:

Frédéric Calon, Ph.D.

Centre de recherche du CHU de Québec – Université Laval

2705, Boulevard Laurier, Room T2-67

Québec, QC, G1V 4G2, Canada

Tel #: +1(418) 525-4444 ext. 48697

Fax #: +1(418) 654-2761

E-mail: Frederic.Calon@crchul.ulaval.ca

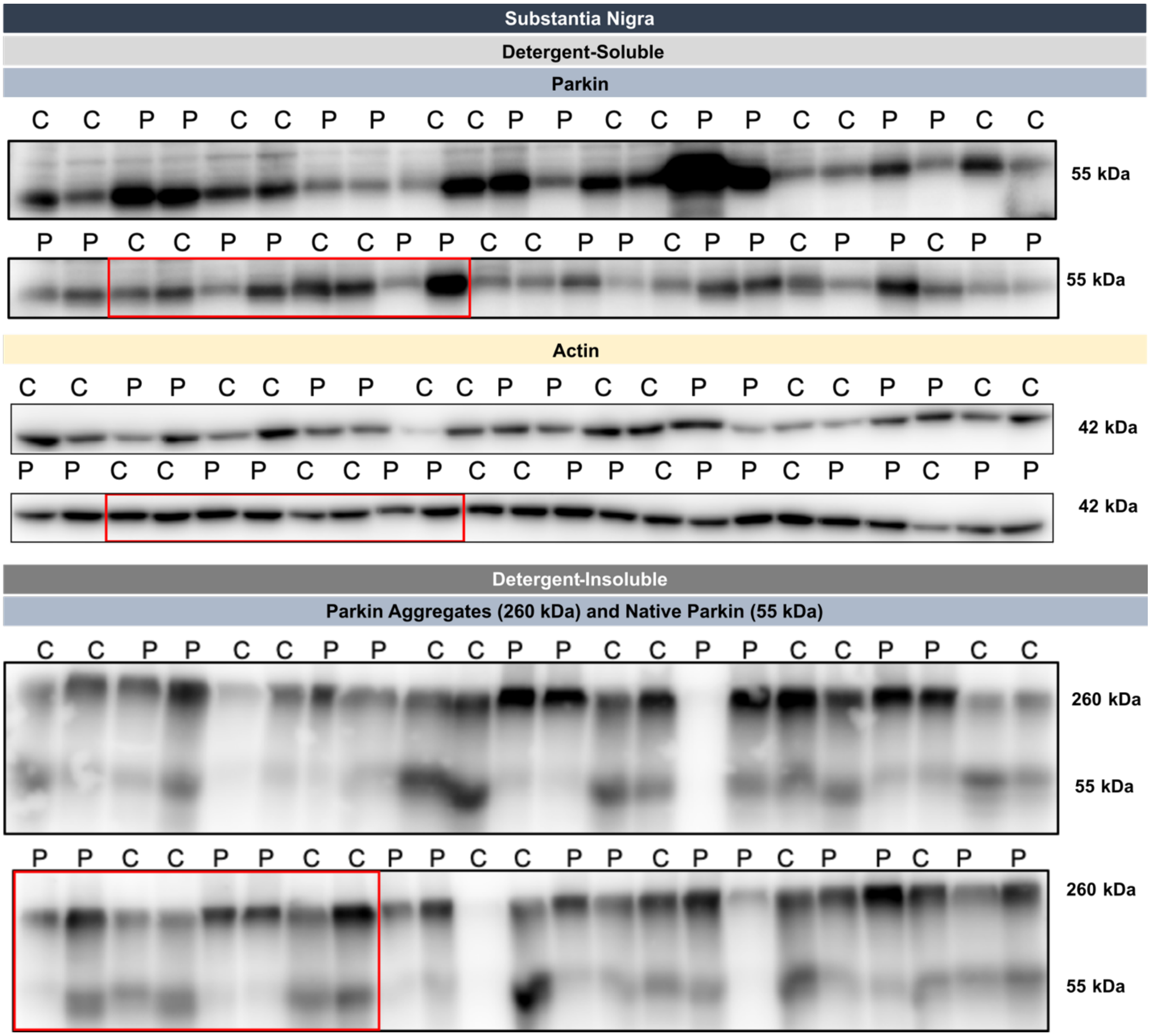

Supplementary Figure 1. Western Blot results for *post-mortem* parkin in the SN. Full WB image of parkin in the detergent-soluble and the detergent-insoluble fractions of the SN. The red rectangles correspond to the representative bands used in the main figures. *Abbreviations: C, control individuals; P, Parkinson's disease patients; WB, Western blot.*

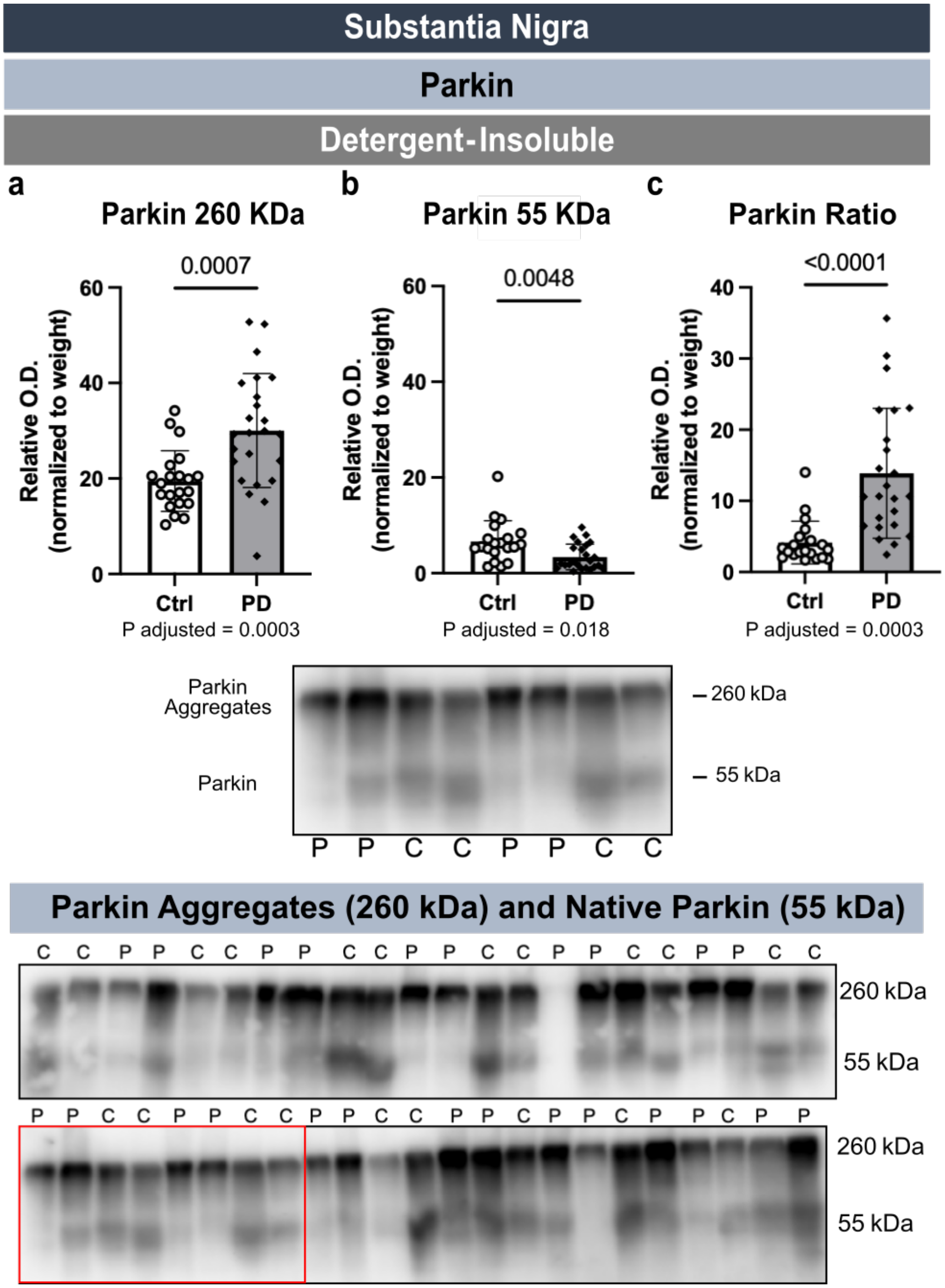

Supplementary Figure 2. Higher HMW and lower monomers of insoluble parkin in the substantia nigra of PD patients, confirmed with a second antibody. Higher levels of detergent-insoluble HMW parkin (260 kDa) (**a**) and lower levels of detergent-insoluble native parkin (55 kDa) (**b**) were detected in the SN of PD patients compared to controls. This leads to a higher detergent-insoluble parkin (260/55) ratio in the SN of the PD group compared to the control group (**c**). Parkin was measured using primary polyclonal antibody #2132 from Cell Signaling targeting C-Terminus region. Full image of the consecutive bands from the samples is also

shown, with red rectangles corresponding to the representative bands used in the main figures. Statistical analysis: data are represented as mean ± SEM (N= 21 ctrl and 24 PD); P-value from Mann-Whitney tests are shown on top, while P-value after adjustments for age and sex are provided below the graphs. Data in a and b were normalized with SN sample weight (mg). *Abbreviations: Ctrl/C, control individuals; HMW, high molecular weight; PD/P, Parkinson's disease patients; SEM, standard error of the mean; SN, substantia nigra. O.D., Optical Density*.

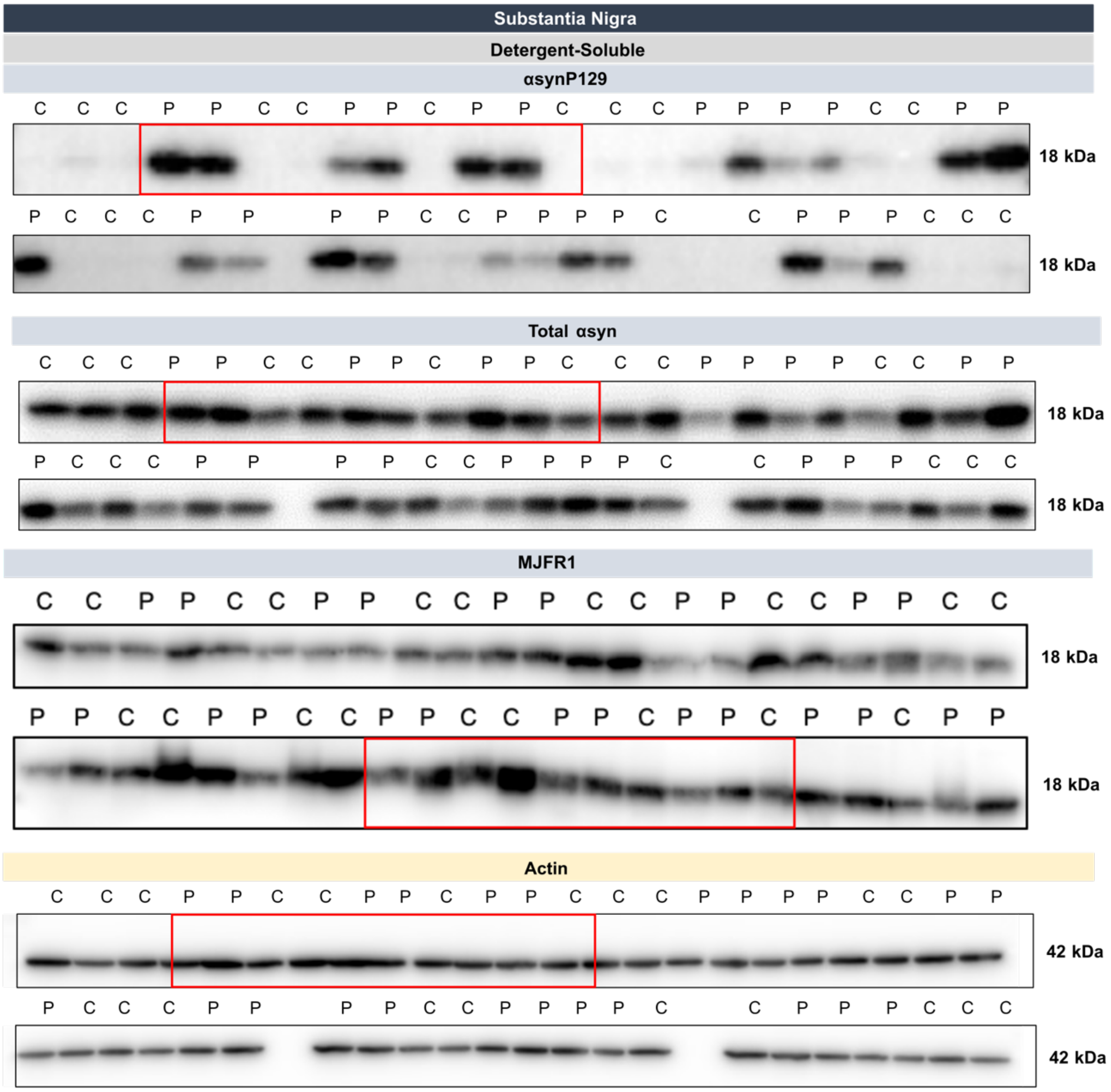

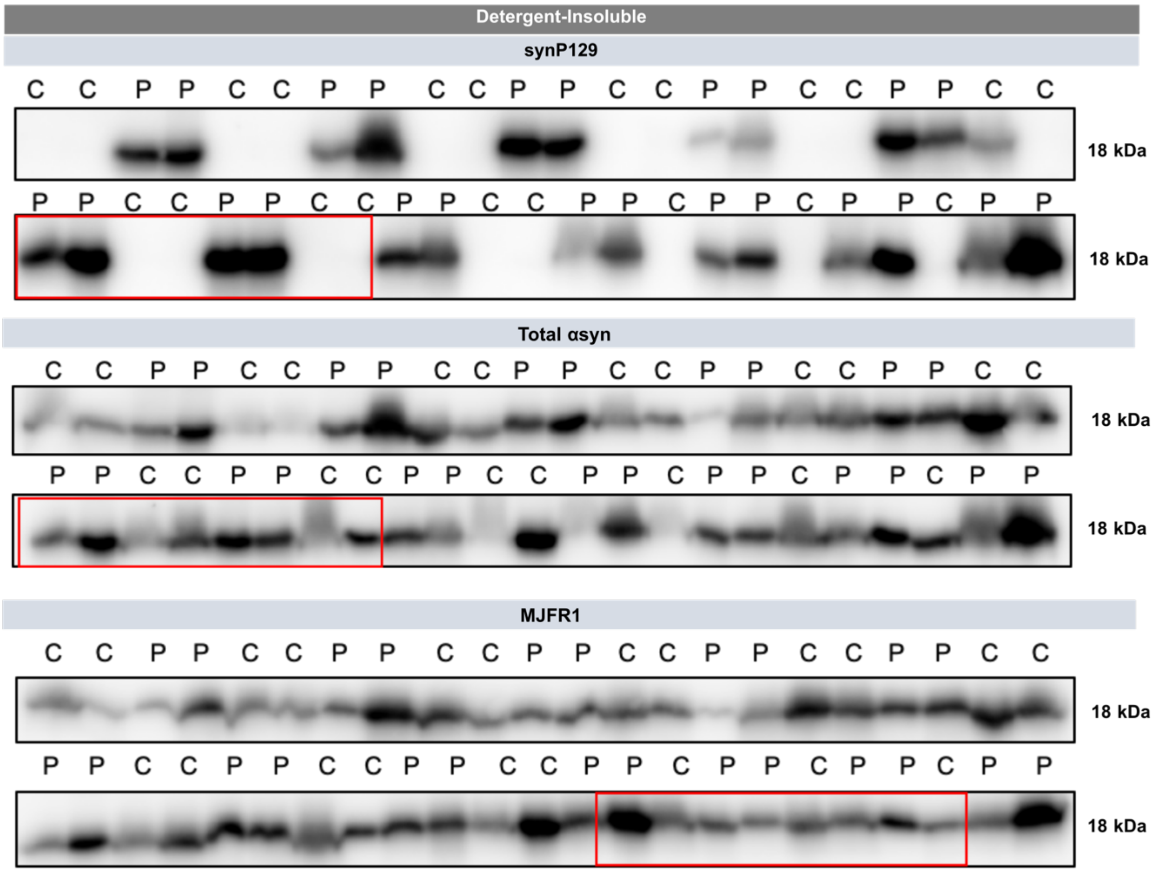

Supplementary Figure 3. Western Blot results for post-mortem αsyn in the SN. Full WB image of αsyn in the detergent-soluble and the detergent-insoluble fractions of the SN. The red rectangles correspond to the representative bands used in the main figures. *Abbreviations: C, control individuals; P, Parkinson's disease patients; asyn, a-synuclein; αsynP129, α-synuclein phosphorylated at serine 129; SN, substantia nigra; WB, Western blot.*

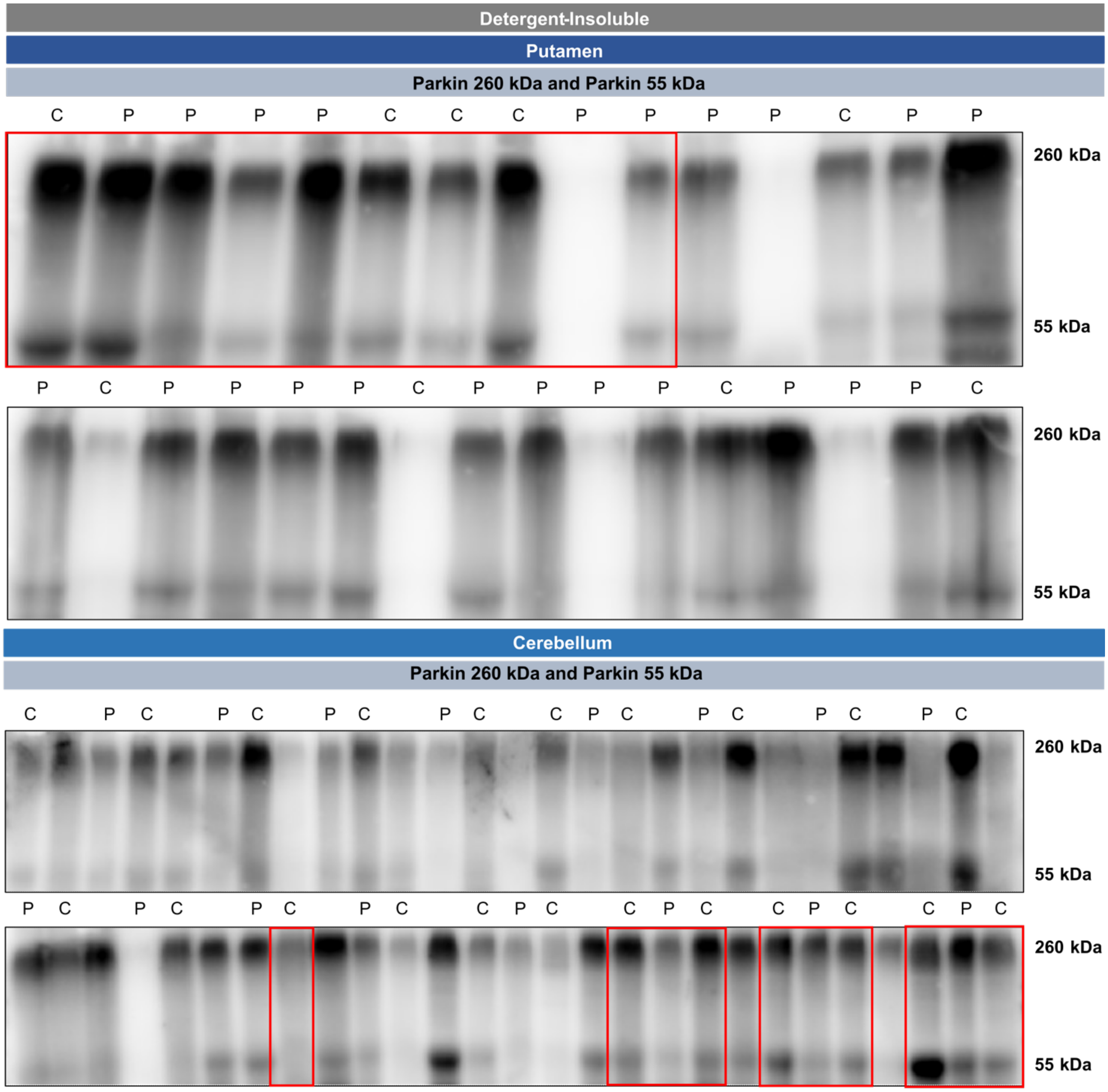

Detergent-Insoluble
Putamen
Parkin 260 kDa and Parkin 55 kDa
C P P P P C C C P P P P C P P
260 kDa
55 kDa
P C P P P P C P P P P C P P P C
260 kDa
55 kDa
Cerebellum
Parkin 260 kDa and Parkin 55 kDa
C P C P C P C P C C P C P C P C P C
260 kDa
55 kDa
P C P C P C P C C P C C P C C P C C P C
260 kDa
55 kDa

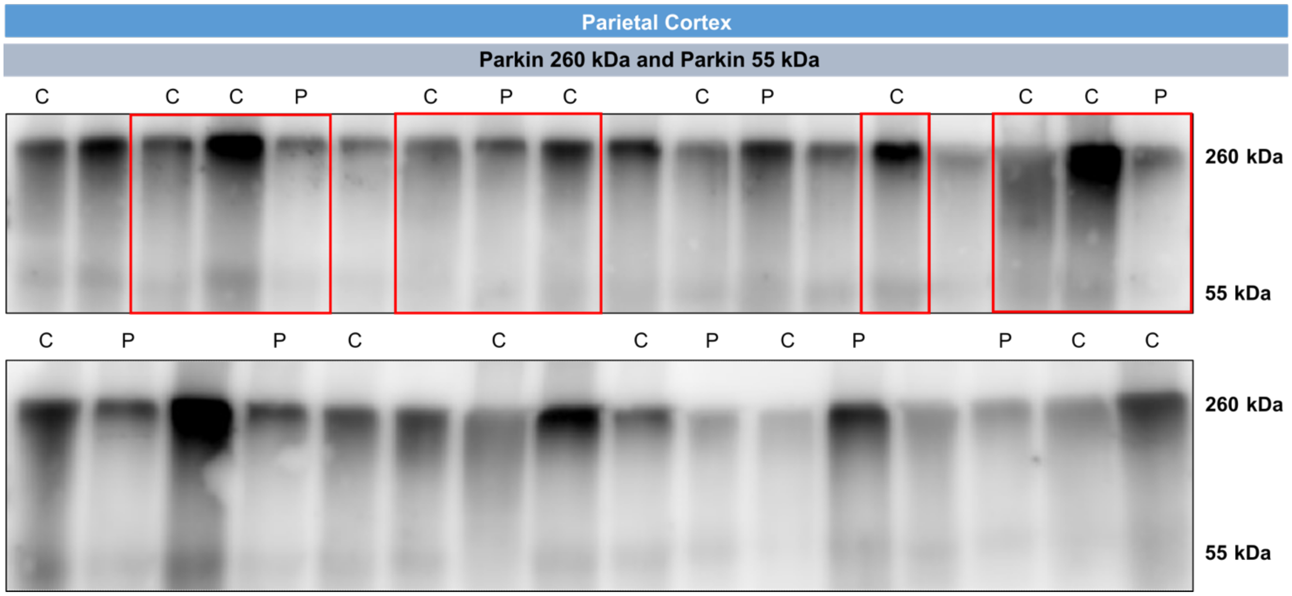

Supplementary Figure 4. Western Blot results for *post-mortem* parkin in the putamen, cerebellum and parietal cortex. Full WB image of parkin in the detergent-insoluble fractions of the putamen, cerebellum and parietal cortex. The red rectangles correspond to the representative bands used in the main figures. For WB in the cerebellum or in the parietal cortex, spaces between controls individuals and PD patients correspond to irrelevant individuals for this study. *Abbreviations: C, control individuals; P, Parkinson's disease patients; WB, Western blot.*

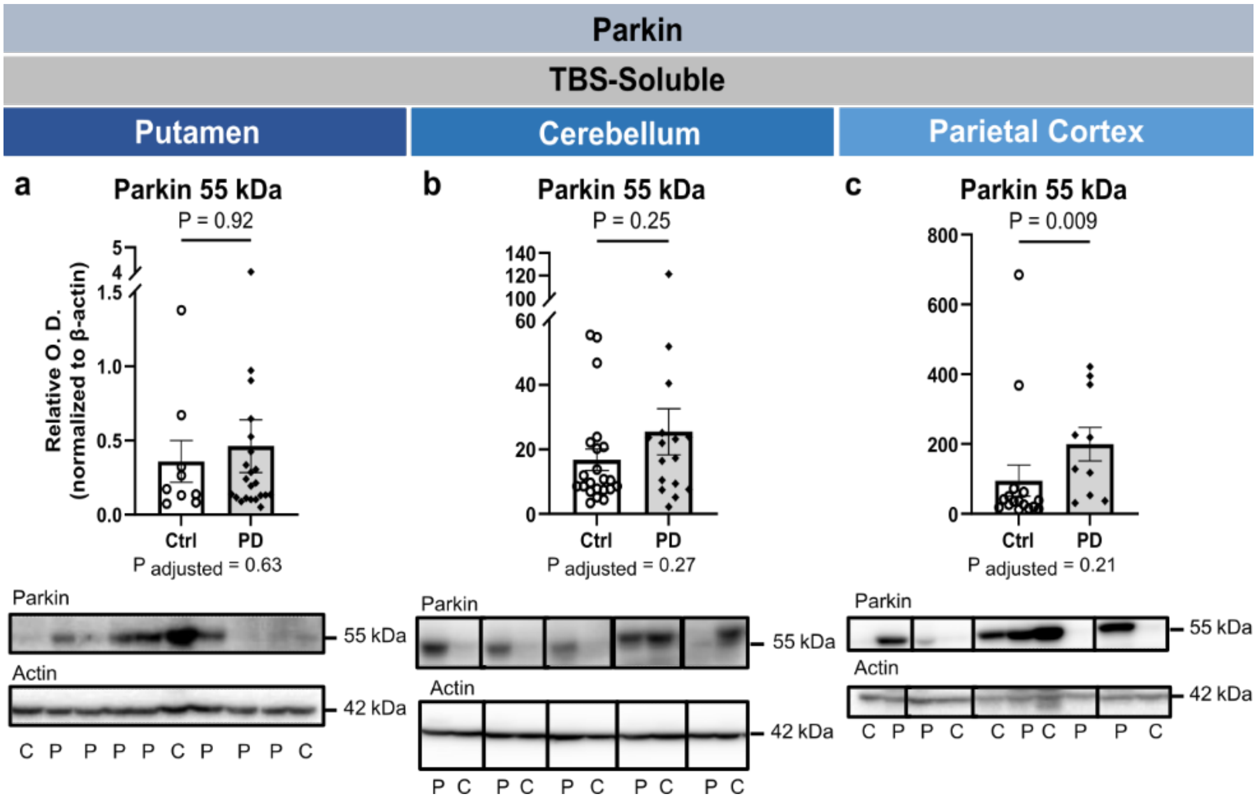

Supplementary Figure 5. Post-mortem levels of TBS-soluble parkin in the putamen, cerebellum and parietal cortex of PD patients. No differences were detected between controls and PD patients for native parkin (55 kDa) in the putamen (**a**) and the cerebellum (**b**) in the TBS-soluble fraction. In the parietal cortex, similar results were obtained after adjustments for age and sex were done (**c**). Statistical analysis: data are represented as mean ± SEM (Putamen, N= 9 ctrl and 22 PD; Cerebellum, N= 22 ctrl and 16 PD; Parietal Cortex, N= 16 ctrl and 10 PD); Mann-Whitney test (**a-c**); the P-value after adjustments for age and sex is provided. *Abbreviations: Ctrl/C, control individuals; PD/P, Parkinson's disease patients; SEM, standard error of the mean; O.D., Optical Density.* Representative WB of bands are shown, where the inserted black vertical line indicates nonconsecutive bands. Full images are shown in Supplementary Figure 5.

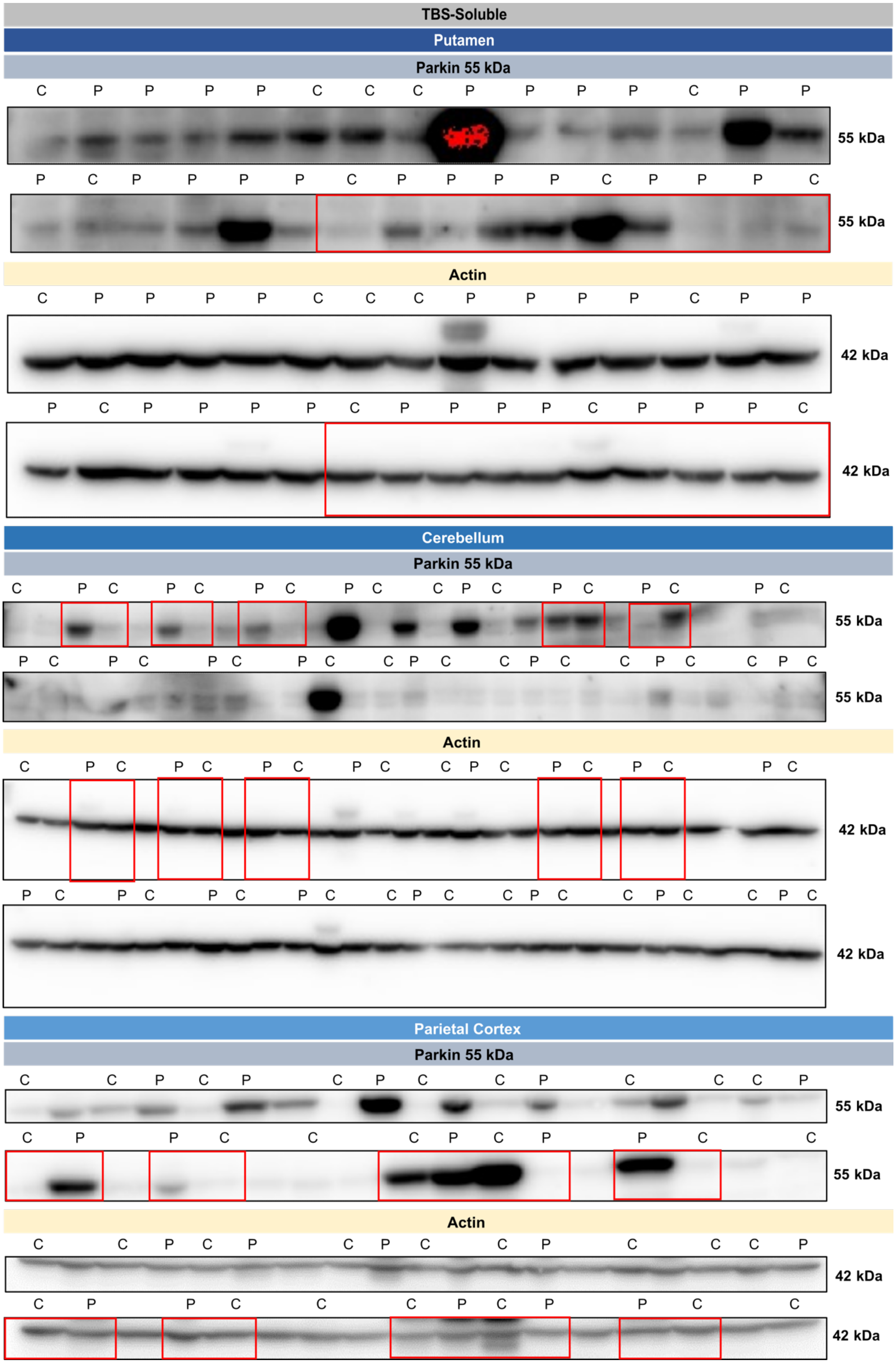
TBS-Soluble
Putamen
Parkin 55 kDa
55 kDa
55 kDa
Actin
42 kDa
42 kDa
Cerebellum
Parkin 55 kDa
55 kDa
55 kDa
Actin
42 kDa
42 kDa
Parietal Cortex
Parkin 55 kDa
55 kDa
55 kDa
Actin
42 kDa
42 kDa

Supplementary Figure 6. Western Blot results for *post-mortem* parkin in the putamen, cerebellum and parietal cortex of the TBS-soluble fraction. Full WB image of parkin in the TBS-soluble fractions of the putamen, cerebellum and parietal cortex. The red rectangles correspond to the representative bands used in the main figures. For WB in the cerebellum or in the parietal cortex, spaces between control individuals and PD patients correspond to irrelevant individuals for this study. *Abbreviations: C, control individuals; P, Parkinson's disease patients; WB, Western blot.*

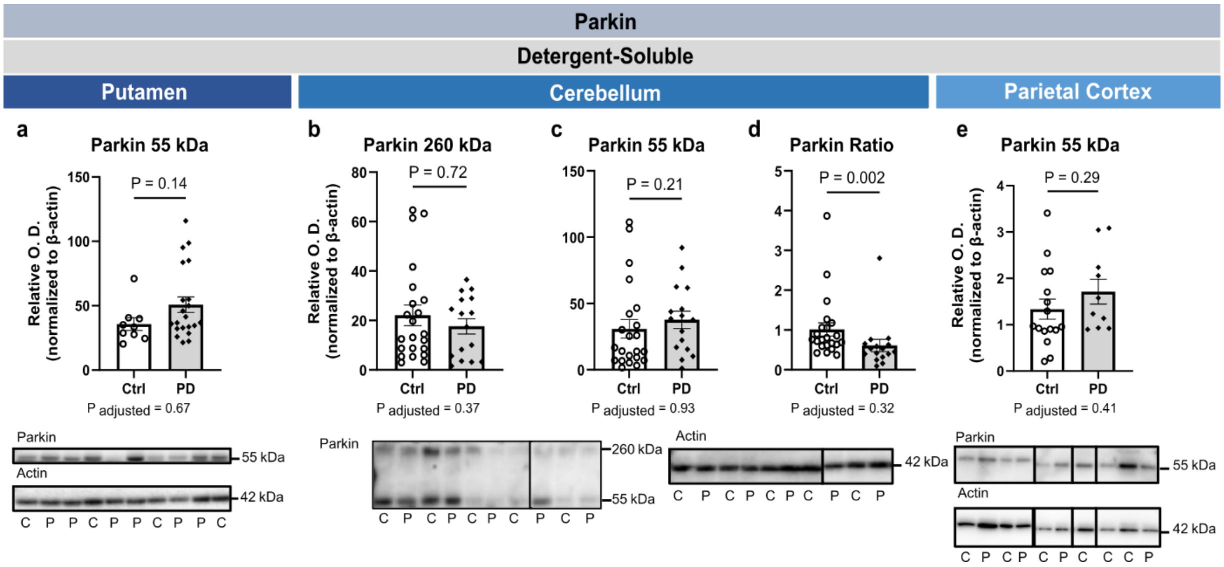

Supplementary Figure 7. Post-mortem levels of detergent-soluble parkin in the putamen, cerebellum and parietal cortex of PD patients. No differences were detected between controls and PD patients for native parkin (55 kDa) in detergent-soluble fractions of the putamen (**a**). In the cerebellum, differences in parkin aggregates (260 kDa) (**b**), native parkin (55 kDa) (**c**) and parkin ratio (260/55) levels (**d**) were nonsignificant between the two groups after adjustments for age and sex. In the parietal cortex, similar results for native parkin (55 kDa) were obtained after adjustments for age and sex were done (**e**). Statistical analysis: data are represented as mean ± SEM (Putamen, N= 9 ctrl and 21 PD; Cerebellum, N= 22 ctrl and 16 PD; Parietal Cortex, N= 16 ctrl and 9 PD); Mann-Whitney test (**a-d**); unpaired student T test (**e**); the P-value after adjustments for age and sex is provided. *Abbreviations: Ctrl/C, control individuals; PD/P, Parkinson's disease patients; SEM, standard error of the mean; O.D., Optical Density.* Representative WB of bands are shown, where the inserted black vertical line indicates nonconsecutive bands. Full images are shown in Supplementary Figure 7.

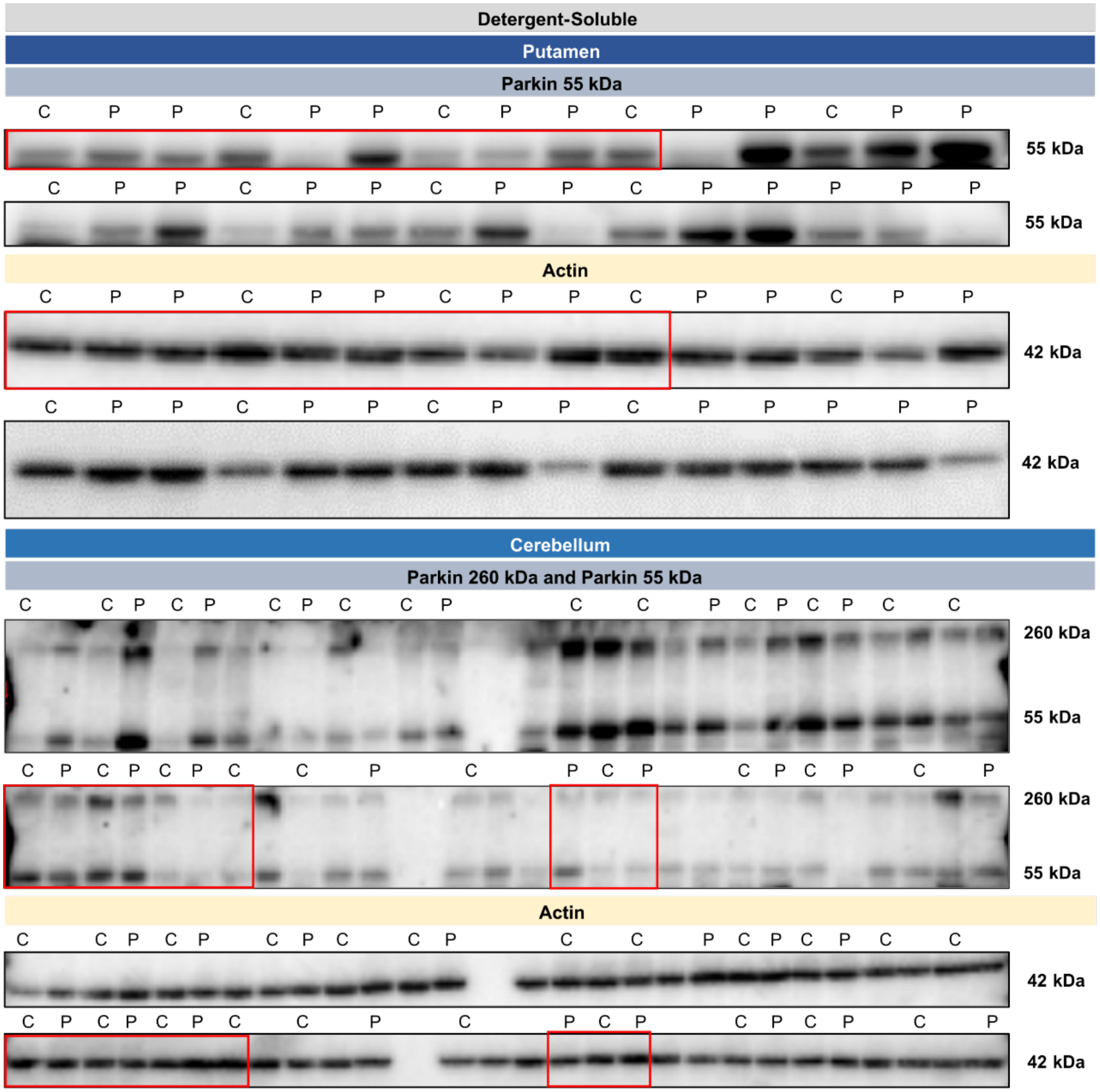
Detergent-Soluble
Putamen
Parkin 55 kDa
C P P C P P C P P C P P C P P
55 kDa
C P P C P P C P P C P P P P P
55 kDa
Actin
C P P C P P C P P C P P C P P
42 kDa
C P P C P P C P P C P P P P P
42 kDa
Cerebellum
Parkin 260 kDa and Parkin 55 kDa
C C P C P C P C C P C C P C P C P C C
260 kDa
55 kDa
C P C P C P C C P C P C P C P C P C P
260 kDa
55 kDa
Actin
C C P C P C P C C P C C P C P C P C C
42 kDa
C P C P C P C C P C P C P C P C P C P
42 kDa

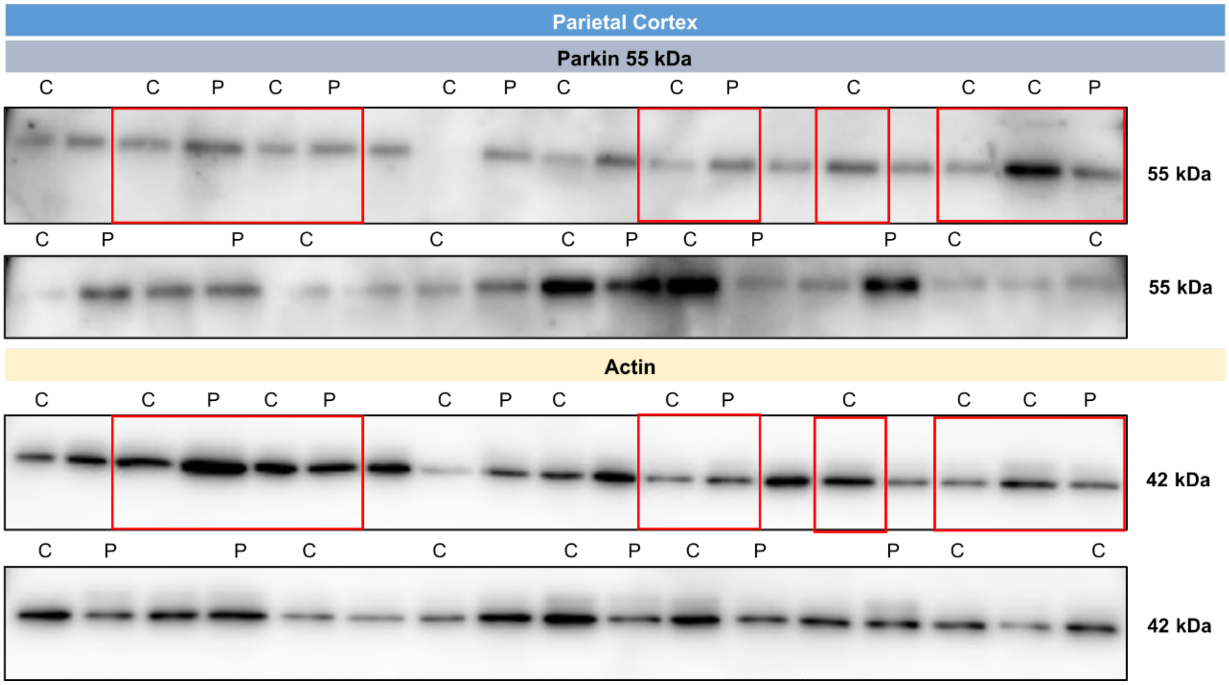

Supplementary Figure 8. Western Blot results for *post-mortem* parkin in the putamen, cerebellum and parietal cortex of the detergent-soluble fraction. Full WB image of parkin in the detergent-soluble fractions of the putamen, cerebellum and parietal cortex. The red rectangles correspond to the representative bands used in the main figures. For WB in the cerebellum or in the parietal cortex, spaces between control individuals and PD patients correspond to irrelevant individuals for this study. *Abbreviations: C, control individuals; P, Parkinson's disease patients; WB, Western blot.*

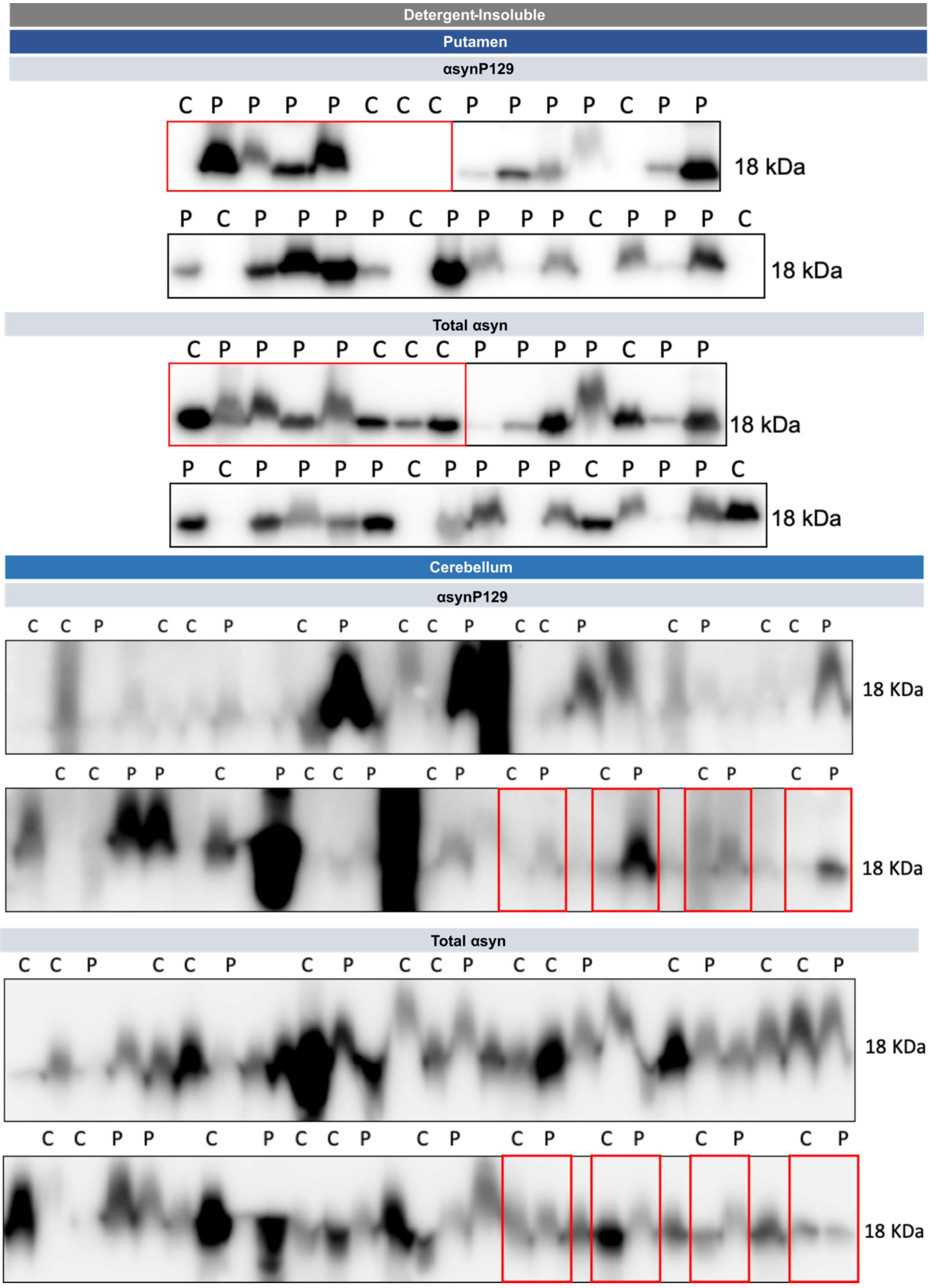
Detergent-Insoluble
Putamen
αsynP129
C P P P P C C C P P P P C P P
18 kDa
P C P P P P C P P P P C P P P C
18 kDa
Total αsyn
C P P P P C C C P P P P C P P
18 kDa
P C P P P P C P P P P C P P P C
18 kDa
Cerebellum
αsynP129
C C P C C P C P C C P C C P C P C C P
18 KDa
C C P P C P C C P C P C P C P C P C P
18 KDa
Total αsyn
C C P C C P C P C C P C C P C P C C P
18 KDa
C C P P C P C C P C P C P C P C P C P
18 KDa

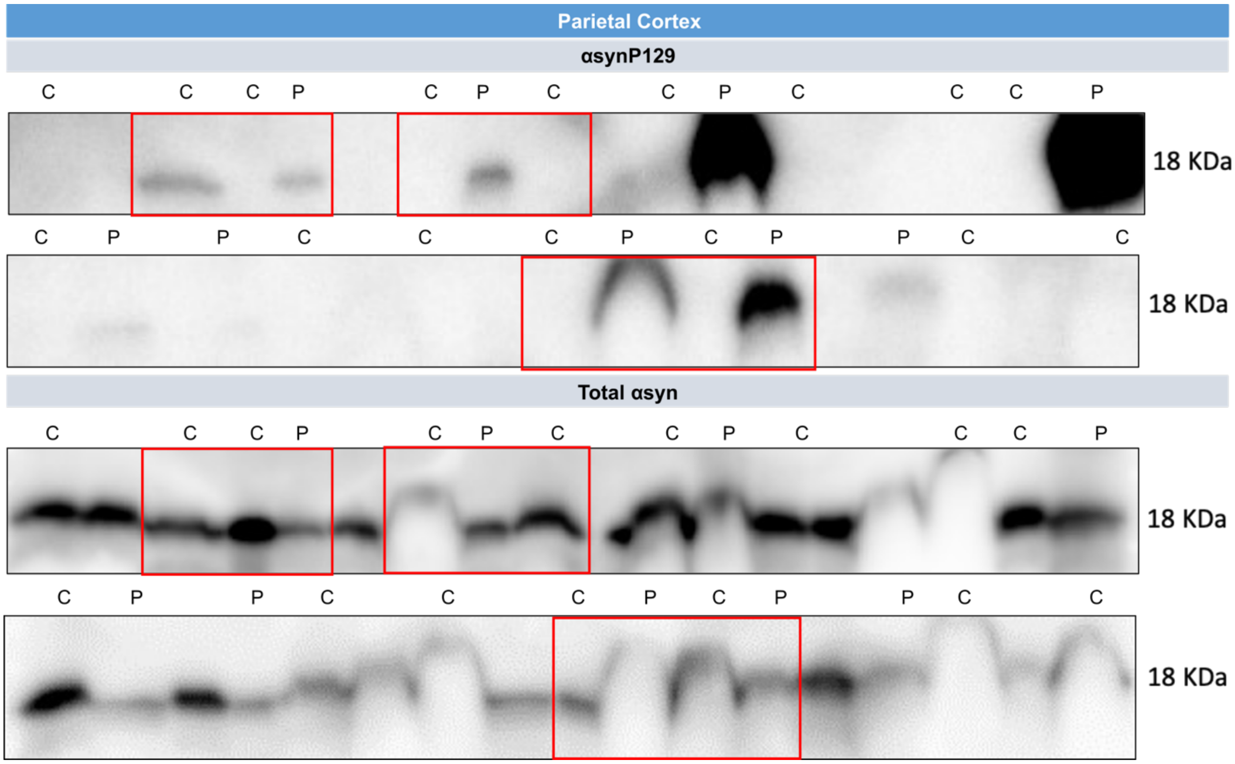

Supplementary Figure 9. Western Blot results for *post-mortem* αsyn in putamen, cerebellum and parietal cortex of the detergent-insoluble fraction. Full WB image of αsyn in the detergent-insoluble fractions of the putamen, cerebellum and parietal cortex. The red rectangles correspond to the representative bands used in the main figures. For WB in the cerebellum or in the parietal cortex, spaces between controls individuals and PD patients correspond to irrelevant individuals for this study. *Abbreviations: C, control individuals; P, Parkinson's disease patients; asyn, a-synuclein; αsynP129, α-synuclein phosphorylated at serine 129; WB, Western blot.*

A0A669KBE3 (100 %), 39 698,5 Da
Parkin RBR E3 ubiquitin protein ligase (Fragment) OS=Homo sapiens OX=9606 GN=PRKN PE=1 SV=1
5 exclusive unique peptides, 5 exclusive unique spectra, 5 total spectra, 77/362 amino acids (21 % coverage)

```
X V F V R F N S S H   G F P V E V D S D T   S I F Q L K E V V A   K R Q G V P A D Q L   R V I F A G K E L R   N D W T V Q N C D L
D Q Q S I V H I V Q   R P W R K G Q E M N   A T G G D D P R N A   A G G C E R E P Q S   L T R V D L S S S V   L P G D S V G L A V
I L H T D S R K D S   P P A G S P A G R S   I Y N S F Y V Y C K   G P C Q R V Q P G K   L R V Q C S T C R Q   A T L T L T Q G P S
C W D D V L I P N R   M S G E C Q S P H C   P G T S A E F F F K   C G A H P T S D K E   T S V A L H L I A T   N S R N I T C I T C
T D V R S P V L V F   Q C N S R H V I C L   D C F H L Y C V T R   L N D R Q F V H D P   Q L G Y S L P C V A   G C P N S L I K E L
H H F R I L G E E Q   Y N R Y Q Q Y G A E   E C V L Q M G G V L   C P R P G C G A G L   L P E P D Q R K V T   C E G G N G L G C G
V S
```

Supplementary Figure 10. Proteomic identification of Parkin in excised Western blot bands. Peptides identified by LC–MS/MS were mapped onto the Parkin protein sequence (UniProt A0A669KBE3), revealing five unique peptides corresponding to 21% sequence coverage. Detected peptides spanned multiple regions of the protein, supporting confident identification. Image generated by Scaffold software Version 5.2.

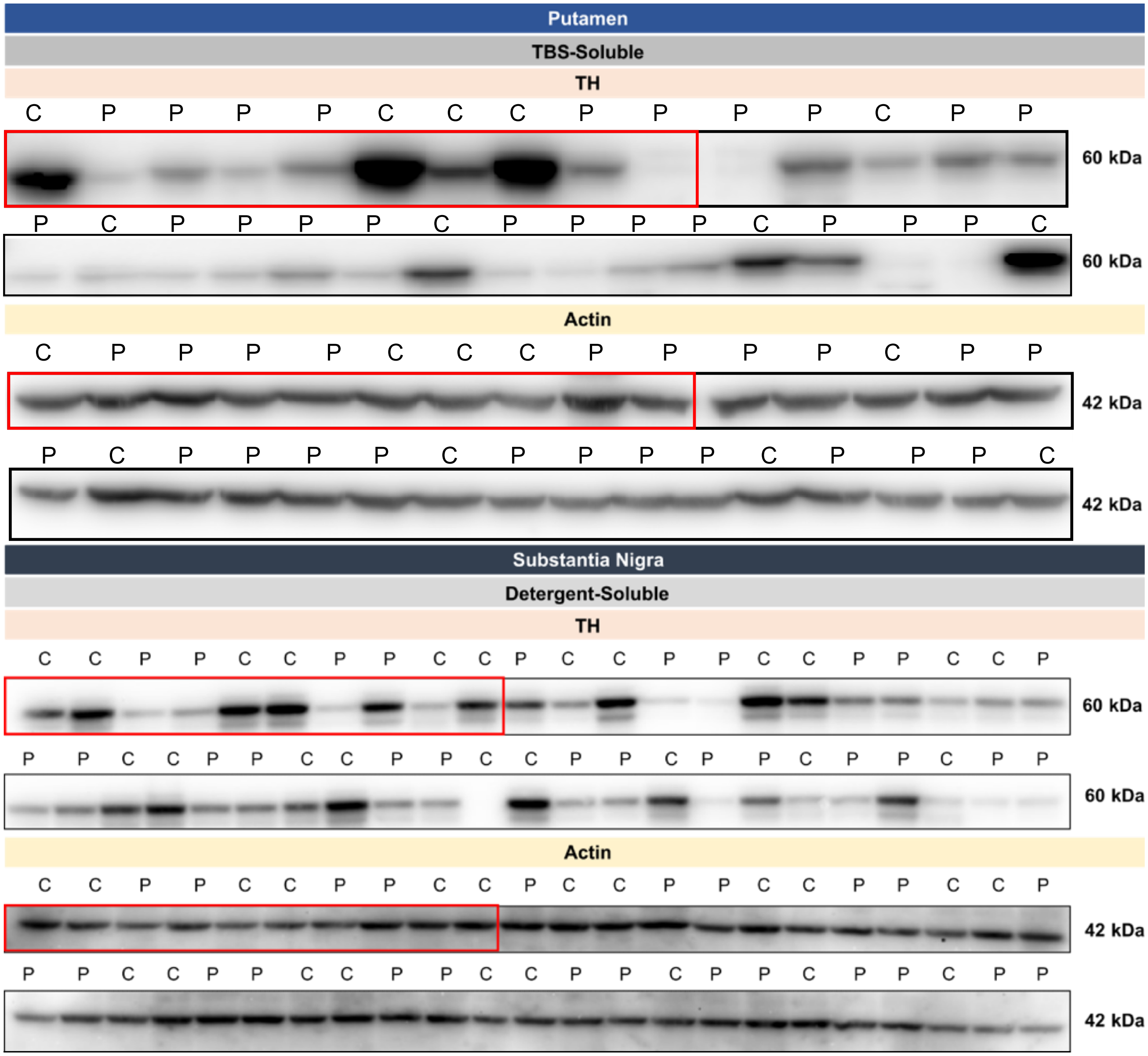

Supplementary Figure 11. Western Blot results for *post-mortem* TH in putamen and substantia nigra. Full WB image of TH in the TBS-soluble fraction of the putamen and in the total fraction of the SN. The red rectangles correspond to the representative bands used in the main figures. *Abbreviations: C, control individuals; P, Parkinson's disease patients; TH, tyrosine hydroxylase; SN, substantia nigra; TBS, tris-buffered saline; WB, Western blot.*

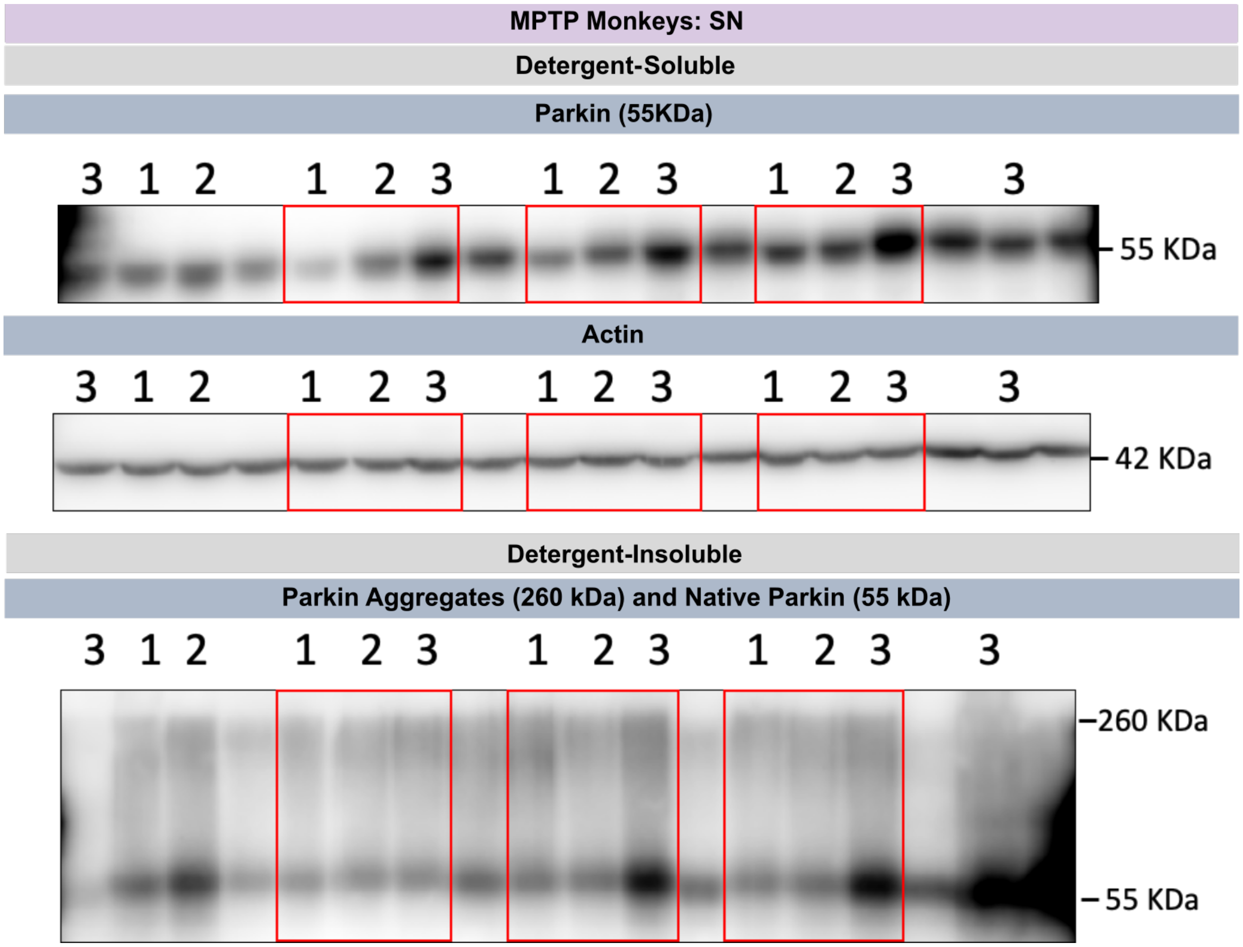

Supplementary Figure 12. Western Blot results for *post-mortem* parkin in the SN of MPTP non-human primates. Full WB image of parkin in the detergent-soluble and the detergent-insoluble fraction of the SN of MPTP non-human primates. The red rectangles correspond to the representative bands used in the main figures. Spaces between groups of monkeys correspond to irrelevant monkeys for this study. Legend for MPTP monkeys WB: 1, intact; 2, MPTP; 3, MPTP+DOPA. *Abbreviations: MPTP, 1-methyl-4-phenyl-1,2,3,6-tetrahydropyridine; DOPA, levodopa; SN, substantia nigra; WB, Western blot.*

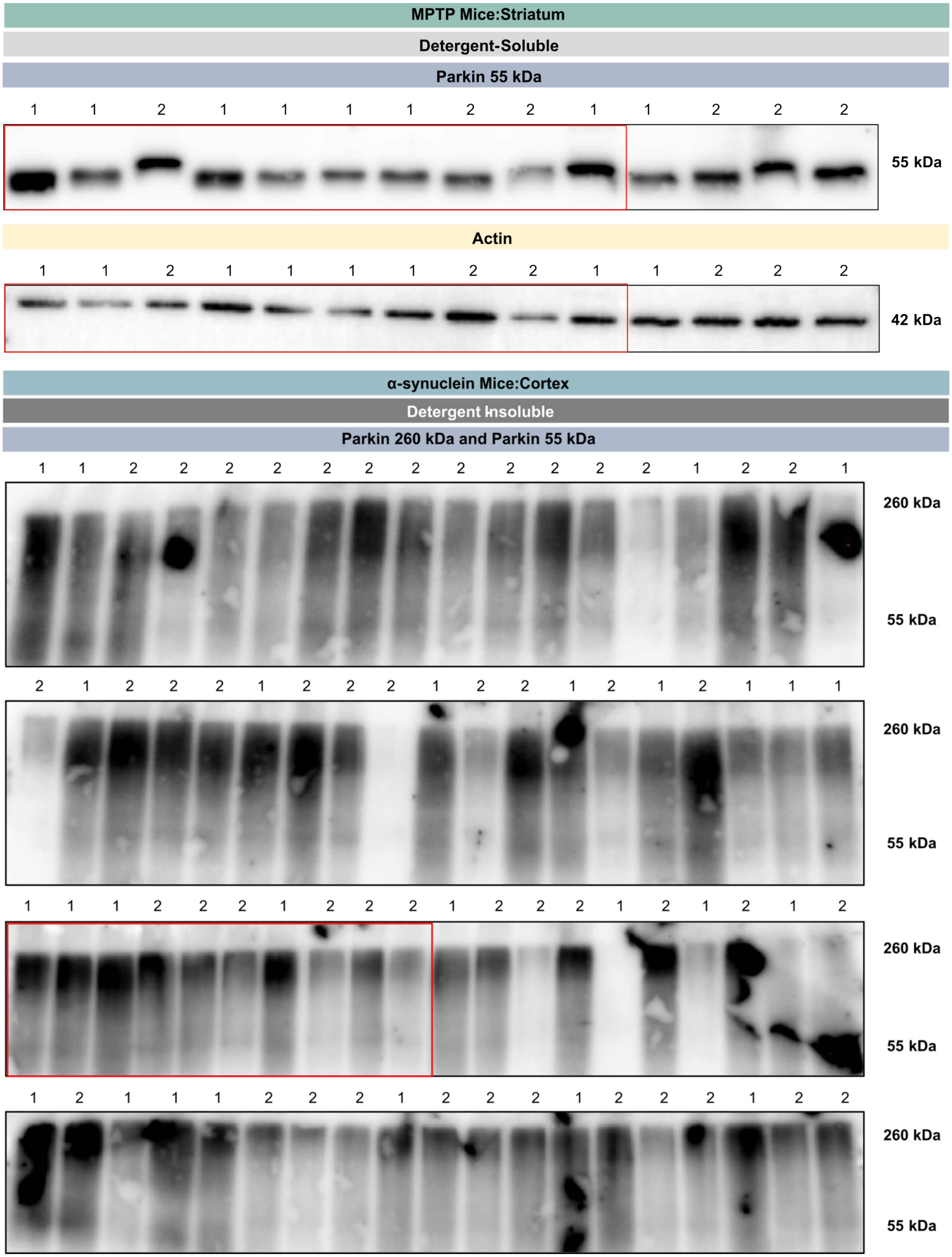
MPTP Mice:Striatum
Detergent-Soluble
Parkin 55 kDa
1 1 2 1 1 1 1 2 2 1 1 2 2 2
55 kDa
Actin
1 1 2 1 1 1 1 2 2 1 1 2 2 2
42 kDa
α-synuclein Mice:Cortex
Detergent Insoluble
Parkin 260 kDa and Parkin 55 kDa
1 1 2 2 2 2 2 2 2 2 2 2 2 2 1 2 2 1
260 kDa
55 kDa
2 1 2 2 2 1 2 2 2 1 2 2 1 2 1 2 1 1 1
260 kDa
55 kDa
1 1 1 2 2 2 1 2 2 2 1 2 2 2 1 2 1 2 1 2
260 kDa
55 kDa
1 2 1 1 1 2 2 2 1 2 2 2 1 2 2 2 1 2 2
260 kDa
55 kDa

Supplementary Figure 13. Western Blot results for parkin in striatum of MPTP mice and in cortex of α-synuclein mice. Full WB image of α-syn in the detergent-soluble fraction of the striatum of MPTP mice and in the detergent-insoluble fraction of the cortex of mice. The red rectangles correspond to the representative bands used in the main figures. Legend for MPTP mice WB: 1, saline; 2, MPTP. Legend for αsyn mice WB: 1, NonTg; 2, Tg (αsyn). *Abbreviations: αsyn, α-synuclein; MPTP, 1-methyl-4-phenyl-1,2,3,6-tetrahydropyridine; NonTg, nontransgenic; Tg, transgenic; WB, Western blot.*

Characteristics	Controls	PD	Statistical Analysis
N	21	24	----
Men/Women	11/10	15/9	C; $\chi2 = 0.47$; p=0.49
Mean Age at Death (years)	75.3 (1.6)	80.5 (1.6)	STT, p=0.011<br>MW, p=0.021
Age of Onset (years)	----	67.7 (2.0)	----
Disease Duration (years)	----	12.6 (1.2)	----
Freezing of Gait (FOG) (n)	----	16/24	----
Age at levodopa Initiation (years)	----	70.5 (2.2)	----
First levodopa Dose (mg)	----	360.4 (43.2)	----
Last levodopa Dose (mg)	----	681.3 (80.6)	----
Levodopa Induced Motor Complications (LIC) No/Yes (n)	----	7/17	----
Dyskinesia Duration (months)	----	52.8 (9.4)	----
Postmortem Interval (hours)	17.8 (1.6)	17.6 (2.3)	STT, p=0.92
Full Brain Weight (g)	1276.25 (63.3)	1300.5 (43.8)	STT, p=0.71
Braak Score 0/1-2/2/3-4/5/5-6 (n)	12/0/0/0/0/1	16/1/3/2/0/0	----
H&Y Stage 0/2/2.5/3/3.5/4/4.5/5 (n)	12/0/1/0/0/0/0/0	0/2/3/3/2/2/4/8	----
SN Lewy Bodies (No/Yes, n)	21/0	0/24	----

Supplementary Table 1. Clinical and demographic for the control and PD groups. The *postmortem* diagnosis was performed by a neuropathologist for each patient and the clinical assessments were accomplished by AHR and AR[2,3]. The values are presented as means (SD). Statistical analyses were performed using Contingency (C), Student unpaired t-test (STT) or Mann-Whitney tests (MW). *Abbreviations: PD, Parkinson's disease patients; C, contingency;*

H&Y, Hoehn and Yahr scale; MW, Mann-Whitney; ROD, relative optical density; SD, standard deviations; SN, substantia nigra; STT, Student unpaired t-test.

1 Morissette, M., Bourque, M., Tremblay, M. & Di Paolo, T. Prevention of L-Dopa-Induced Dyskinesias by MPEP Blockade of Metabotropic Glutamate Receptor 5 Is Associated with Reduced Inflammation in the Brain of Parkinsonian Monkeys. *Cells* **11** (2022). https://doi.org:10.3390/cells11040691

2 Rajput, A. H. Contributions of human brain biochemical studies to movement disorders. *Parkinsonism Relat Disord* **8**, 425-431 (2002). https://doi.org:10.1016/s1353-8020(02)00026-3

3 Rajput, A. H. & Rajput, A. Saskatchewan movement disorders program. *Can J Neurol Sci* **42**, 74-87 (2015). https://doi.org:10.1017/cjn.2015.13

The ARRIVE Essential 10: Compliance Questionnaire

Use this questionnaire to evaluate how well a manuscript complies with the ARRIVE Essential 10. It can be applied to any manuscript describing comparative experiments in living animals, by assessors such as journal staff, editors, or peer reviewers.

Item	Question(s)	Answers
1 Study Design	Are all experimental and control groups clearly identified?	☒ Yes, for at least one experiment<br>☐ No
	Is the experimental unit (e.g. an animal, litter or cage of animals) clearly identified?	☒ Yes, for at least one experiment<br>☐ No
2 Sample Size	Is the exact number of experimental units in each group at the start of the study provided (e.g. in the format '*n*=')?	☒ Yes, for at least one experiment<br>☐ No
	Is the method by which the sample size was chosen explained?	☒ Yes, for at least one experiment<br>☐ No
3 Inclusion & Exclusion Criteria	Are the criteria used for including and excluding animals, experimental units, or data points provided?	☒ Yes, for at least one experiment<br>☐ No
	Are any exclusions of animals, experimental units, or data points reported, or is there a statement indicating that there were no exclusions?	☒ Yes, for at least one analysis<br>☐ No
4 Randomisation	Is the method by which experimental units were allocated to control and treatment groups described?	☒ Yes, for at least one experiment<br>☐ No
5 Blinding	Is it clear whether researchers were aware of, or blinded to, the group allocation at any stage of the experiment or data analysis?	☒ Yes, for at least one experiment<br>☐ No
6 Outcome Measures	For all experimental outcomes presented, are details provided of exactly what parameter was measured?	☒ Yes, for at least one experiment<br>☐ No
7 Statistical Methods	Is the statistical approach used to analyse each outcome detailed?	☒ Yes, for at least one analysis<br>☐ No
	Is there a description of any methods used to assess whether data met statistical assumptions?	☒ Yes, for at least one analysis<br>☐ No<br>☐ Not applicable
8 Experimental Animals	Are all species of animal used specified?	☒ Yes, for at least one experiment<br>☐ No
	Is the sex of the animals specified?	☒ Yes, for at least one experiment<br>☐ No<br>☐ Not applicable to species
	Is at least one of age, weight or developmental stage of the animals specified?	☒ Yes, for at least one experiment<br>☐ No
9 Experimental Procedures	Are both the timing and frequency with which procedures took place specified?	☒ Yes, for at least one experiment<br>☐ No
	Are details of acclimatisation periods to experimental locations provided?	☒ Yes, for at least one experiment<br>☐ No
10 Results	Are descriptive statistics for each experimental group provided, with a measure of variability (e.g. mean and SD, or median and range)?	☒ Yes, for at least one experiment<br>☐ No<br>☐ Not applicable to the type of data collected
	Is the effect size and confidence interval provided?	☒ Yes, for at least one experiment<br>☐ No<br>☐ Not applicable to the type of analysis used

ARRIVE

NC3Rs National Centre for the Replacement Refinement & Reduction of Animals in Research

Notes on questionnaire design

The ARRIVE guidelines are a useful resource for authors preparing manuscripts describing animal research, and also provide a framework to evaluate the transparency of those manuscripts. To assess reporting quality, numerous studies have in the past sought to operationalise reporting guidelines (including ARRIVE). Typically, this involves scoring a manuscript's degree of compliance with guideline items in a binary fashion (e.g. an item is either not reported or reported) [1-3], a graded fashion (e.g. not, partially, or completely reported) [4,5], or a combination of the two [6].

This questionnaire has been designed to be as concise and user-friendly as possible. The number of questions used to assess a manuscript's compliance has been kept to a minimum, and in most cases each question is designed to be answered in a binary fashion. Compliance with some Essential 10 sub-items is inherently impossible to judge in this way, instead requiring a subjective judgement on the level of detail provided. For this reason, not all sub-items are represented by a question in this questionnaire.

To facilitate binary answers, it has been necessary to identify the minimum information in a manuscript sufficient to comply with each question. The strengths of this approach include the relatively short length of the questionnaire (and the correspondingly low time burden of using it), and the avoidance of ambiguity that would arise from a graded answering system, in which an intermediate score (e.g. 'partially/insufficiently reported') could denote a number of distinct deficiencies in compliance with an item (e.g. either only part of the item was complied with, or only the reporting of some experiments in the manuscript complied with the item.)

Limitations of this approach centre on the necessity to identify the minimum information sufficient to comply with each question. In some cases, this has resulted in questions that require a guideline sub-item's criteria to have been fulfilled in the reporting of only one experiment in a manuscript. As a result, not all experiments in a manuscript may be described in a way that fulfils that criterion, despite the manuscript being considered to comply with the guidelines overall.

References

1. Hair *et al* (2020). *Res Integ Peer Rev*. doi: 10.1186/s41073-019-0069-3
2. Tihanyi *et al* (2019). *J Surg Res*. doi: 10.1016/j.jss.2018.10.038
3. Zhao *et al* (2020). *BMC Vet Res*. doi: 10.1186/s12917-020-02664-1
4. Han *et al* (2017). *Plos One*. doi: 10.1371/journal.pone.0183591
5. Chatzimanouil *et al* (2019). *J Am Soc Nephrol*. doi: 10.1681/ASN.2018050515
6. Leung *et al* (2018). *Plos One*. doi: 10.1371/journal.pone.0197882
